# Evolution of Therapeutic Antibodies, Influenza Virus Biology, Influenza, and Influenza Immunotherapy

**DOI:** 10.1155/2018/9747549

**Published:** 2018-05-28

**Authors:** Urai Chaisri, Wanpen Chaicumpa

**Affiliations:** ^1^Department of Tropical Pathology, Faculty of Tropical Medicine, Mahidol University, Bangkok, Thailand; ^2^Center of Research Excellence on Therapeutic Proteins and Antibody Engineering, Department of Parasitology, Faculty of Medicine Siriraj Hospital, Mahidol University, Bangkok, Thailand

## Abstract

This narrative review article summarizes past and current technologies for generating antibodies for passive immunization/immunotherapy. Contemporary DNA and protein technologies have facilitated the development of engineered therapeutic monoclonal antibodies in a variety of formats according to the required effector functions. Chimeric, humanized, and human monoclonal antibodies to antigenic/epitopic myriads with less immunogenicity than animal-derived antibodies in human recipients can be produced* in vitro*. Immunotherapy with ready-to-use antibodies has gained wide acceptance as a powerful treatment against both infectious and noninfectious diseases. Influenza, a highly contagious disease, precipitates annual epidemics and occasional pandemics, resulting in high health and economic burden worldwide. Currently available drugs are becoming less and less effective against this rapidly mutating virus. Alternative treatment strategies are needed, particularly for individuals at high risk for severe morbidity. In a setting where vaccines are not yet protective or available, human antibodies that are broadly effective against various influenza subtypes could be highly efficacious in lowering morbidity and mortality and controlling unprecedented epidemic/pandemic. Prototypes of human single-chain antibodies to several conserved proteins of influenza virus with no Fc portion (hence, no ADE effect in recipients) are available. These antibodies have high potential as a novel, safe, and effective anti-influenza agent.

## 1. Introduction

**Figure 1:**
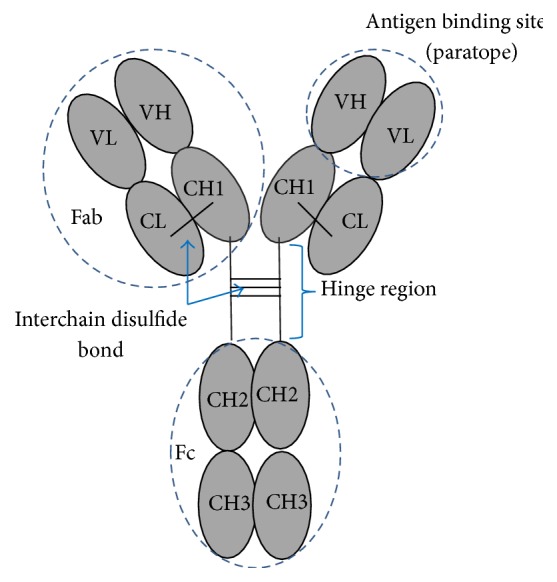
Basic structure of conventional antibody molecule, such as human IgG.

Antibodies are glycoproteins of the immunoglobulin superfamily. Antibodies are produced by plasma cells which are derived from differentiated B lymphocytes of the immune system in response to foreign substances. The basic structure of an antibody molecule such as human immunoglobulin G (IgG) (Figure 1) consists of the four polypeptide chains: two identical heavy (H) chains and two identical light (L) chains. One light chain (either *κ* or *λ* type) is linked alongside one H chain (*μ*, *δ*, *γ*, *α*, or *ε*), while the two H chains are linked together, also by disulfide bonds. There are two immunoglobulin domains in each L chain that are designated (from N-terminal) variable (VL) and constant (CL) domains. Each H chain contains one variable (VH) domain and 3-4 constant (CH) domains (i.e., CH1–CH3 for IgA, IgD, and IgG, and CH1–CH4 for IgM and IgE). The VL and VH domains form an antigen-binding site (paratope), while the constant part of the molecule determines antibody classes or isotypes (IgM, IgD, IgG, IgA, or IgE) and other biological functions, including complement activation and fixation to cell surface receptors (Fc receptors; FcR). Antibodies provide host resistance to invaders, such as microorganisms, by different mechanisms (antibody-mediated immunity), either alone or* via* cooperation with other humoral and cellular factors of the immune system [1, 2].

Antibodies may be produced by a host's immune system after exposure to an infectious agent or foreign matter, or after vaccination/immunization (actively acquired immunity). Alternatively, an individual may receive antibodies produced from other sources (passive immunization/passively acquired immunity), such as transfer of maternal antibodies to fetus* in utero* through the placenta or to infants* via *colostrum and breast milk (naturally acquired). Ready-made antibodies may be given to a recipient through injection/infusion (artificially acquired). The actively acquired immunity sustains relatively longer (can be life-long) compared to the passive immunity. Besides, the antigen primed-lymphocytes can retain immunological memory which upon reexposure to the same or antigenically related substance will respond at an accelerated rate with a higher magnitude of the response than the previous contact. However, there are many limitations in generating the actively acquired immunity. Usually, there is a time-lapse between the antigen exposure and the emergence of the effective immune response; thus active immunization is not practical for intervening morbidity after exposure to pathogens of short incubation period (such as influenza) or highly toxic substances (*e.g.*, snake venoms, many toxins). Besides, induction of the active immune response depends on several factors including characteristics of antigen/immunogen (immune-dominant* versus* low immunogenic; particulate/aggregate versus soluble; and complex* versus* single/simple subunit) and other attributions, such as dose of the antigen (too high or too low) and route of immunization (parenteral* versus* mucosal); immunological adjuvant used and host factors (genetics, MHC allotypes, age, nutritional status, and immune competency). Not all subjects will respond and acquire protective immunity after antigenic exposure. Maternal antibodies are known to interfere with vaccine immunogenicity in infants. It is also difficult (and sometimes impossible) to induce active immune response against highly toxic substance that the immunogenic dose is higher than the disease causing dose.

Passive transfer of ready-made antibodies provides immediate immunity although for a short duration. Passive immunization had been practiced for prophylaxis, intervention, and treatment of diseases since the late 18th century. The measure was called serum therapy at the time. In 1890, Emil Adolf von Behring and Shibasaburo Kitasato successfully treated diphtheritic children with serum from animals immune to diphtheria [3, 4]. Since then, diphtheria antitoxins and many other antitoxins, for example, antitetanus toxin, antibotulinum toxin, and antistaphylococcal toxic shock syndrome toxin-1 (TSST-1), have been used for specific treatment of the respective entities. The therapeutics may be in the form of refined immunoglobulins for intravenous (IVIG) or intramuscular administrations instead of immune sera [4]. Passive immunization was used also in postexposure prophylaxis for intervention of morbidity [5]. For examples, hepatitis B immune globulin (HBIG) is given to subjects exposed to the hepatitis B virus (HBV) through contaminated needle stick or sexual exposure or to a newborn of infected mother at parturition [5]. A combination of active and passive immunization has been practiced for intervention of rabies in rabid dog bitten subjects whose bite wounds are serious and/or located near to the central nervous system. In this instance, human rabies immune globulins (HRIG) and rabies vaccines are given concomitantly at different sites; the former is for providing immediate immunity (the HRIG is infiltrated around the biting wound and injected intramuscularly) and the latter for eliciting a longer lasting immunity to the virus [5]. Although emergence of sulfonamides and antibiotics in the 1930–1940s led to the use of the drugs for treatment of most bacterial infections; however, antibodies still have their prophylactic and therapeutic applications for many diseases including intoxication/toxemia/envenomation/poison, many viral infections, cancers, autoimmune disorders, cardiovascular diseases, chronic inflammatory diseases, allergy, allograft rejection, and graft* versus* host (GVH) reaction [5–8]. Passive immunization has been considered also as an option for treatment of deliberately released infectious biological agents in bioterrorism (biological weapons) such as anthrax, botulinum, plague, smallpox, and tularemia [9]. Antibodies may be an adjunct of supportive therapy for infections/intoxications that direct acting agents are not available or for infections caused by drug resistant pathogens [10].

## 2. Therapeutic Antibodies

### 2.1. Animal-Derived Polyclonal and Monoclonal Antibodies

Therapeutic polyclonal antibodies (PAbs) obtained from serum/plasma of specifically immunized animals (such as horse, sheep, donkey, camel, goat, and rabbit) have been used in the early days of serum therapy. Nevertheless, adverse side effects including immediate reactions (both IgE and non-IgE mediated), pyrogenicity, and/or delayed serum sickness occur frequently in human recipient [11]. The animal immunization requires repeated and lengthy immunization process before a satisfactory antibody level is reached [12]. Besides, limited amount of the immune serum/plasma (compared to the high demand) is obtained from individual animals at one bleeding time. There is a batch-to-batch variation of the antibody quality as well as a difficulty in eliciting antibodies against low immunogenic but highly toxic substances. Moreover, large animals require large pasture for gazing and roomy shelter. Proper husbandry must be provided in order to keep them in good health and free of infection, particularly zoonosis such as equine encephalitis that may be transmitted to the animal caretakers or the recipients of the antibodies.

Invention of hybridoma technology by Köhler and Milstein in 1975 [13] has abolished some limitations of therapeutic antibody production by animal immunization. Mouse monoclonal antibodies with well-defined target-specificity, high purity, and reproducible quality at the desired amount can be produced by growing an established hybridoma clone (derived from fusion of parental B and mouse myeloma cells)* in vitro*. Monoclonal antibodies from the hybridoma technology have been used extensively in immunoassays [14–16], imaging [17], and passive immunotherapy of infectious and noninfectious diseases [18, 19]. The first therapeutic mouse monoclonal antibody, that is, muromonab, which is IgG2a specific to CD3 on T cells was approved by US-FDA in 1986 for treatment of allograft rejection [20]. Limitations of the mouse monoclonal antibody include requirement of tissue culture facility, strict aseptic techniques, and expensive culture medium. Hybridoma cultures require frequent subcultures due to rapid depletion of nutrients in the growth medium, deposition and accumulation of apoptotic/necrotic cells, and cell cycle arrest (which can stop dividing, known as senescence stimulated by cell-to-cell contact) [21]. The major obstacle in using mouse monoclonal antibodies for human treatments is their immunogenicity. The mouse proteins are foreign to human immune system and human anti-mouse antibody (HAMA) response is elicited which leads to a rapid clearance of the mouse antibody and adverse reactions [22–24]. Besides, murine monoclonal antibodies are relatively inefficient in antibody-dependent cellular cytotoxicity (ADCC) and complement-dependent cytotoxicity (CDC), the activities which are critical for anticancers [25].

### 2.2. Engineered Antibodies

**Figure 2:**
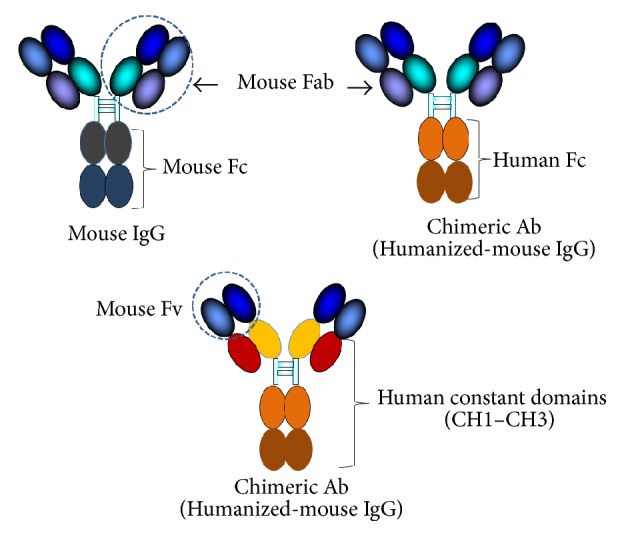
Mouse IgG and humanized-mouse IgG (chimeric antibody). The mouse protein, that is, Fc or the whole constant part, is replaced by the respective human counterpart.

The first attempt to reduce immunogenicity of the therapeutic mouse monoclonal antibody was to replace the mouse Fc fragment or the whole antibody constant regions (CH1–CH3) with the human counterpart by means of genetic manipulations, that is, mutations or engineering [26, 27]. The chimeric antibody (Figure 2) retains the mouse Fab or Fv (VH-VL) fragments with the same epitopic specificity to the original molecule but the immunogenicity to the human immune system is reduced by ≥70% [27, 28]. Retention of the Fc portion in the chimeric molecules is necessary for maintaining the immune effector activities such as Fc-dependent ADCC or binding to cellular receptor for enhancement of phagocytosis, activation of complement, and clearance of immune complexes. The first chimeric human-mouse monoclonal antibody, that is, rituximab, was approved by FDA in 1997. It is mouse IgG1 specific to CD20 for treatment of non-Hodgkin lymphomas [29]. Alternative approach for production of chimeric human-animal antibodies has emerged through the use of humanized-rodent such as OmniRat that carries a chimeric human/rat IgH locus and fully human Ig*κ* or Ig*λ* locus [30].

Mouse antibody fragments including F(ab)′_2_, Fab, and single-chain antibody variable fragment (scFv, which the VH is linked to the VL by a polypeptide) can be used for human therapy when the effector functions of the Fc are not needed. However, these antibody fragments are still immunogenic in human recipients [20, 26]. To obviate this problem, further reduction of the mouse antibody immunogenicity was performed by molecular grafting all antigen-binding loops (complementarity determining regions, CDRs) of the mouse antibody onto the closest human immunoglobulin framework regions (FRs) (humanization process) [31–34]. The humanized-mouse antibody retains the antigenic specificity of the parental molecule (provided that the conformation of the antigen-binding fragment is conserved after humanization, protein purification, and refolding). Alternatively, humanization of murine antibody can be done by replacing some surface-exposed residues of the mouse framework regions with those of the human regions while maintaining the CDRs and core residues of the mouse framework (residues that are important for maintaining the affinity, i.e., canonical structure or regions that contact with the antigen). This process is called “Resurfacing” [35]. The first humanized-monoclonal antibody, antithrombolism, was approved by the US-FDA in 1994 [36]. Nowadays, many of the licensed therapeutic monoclonal antibodies are in the humanized-mouse format ([19, 37], and others).

Production of the humanized-antibodies by CDR grafting or resurfacing is laborious and cumbersome as it has to be done antibody-by-antibody. Sometimes the target binding affinity or specificity of the parental molecule is not maintained. Nowadays, fully human monoclonal antibodies can be produced by using several strategies. Human monoclonal antibodies could be produced from hybrids which were obtained by fusion of either immunized peripheral blood lymphocytes or immune B cells obtained at disease recovery period with human lymphoblastoid or lymphoma cell lines (human hybridomas) [38]. Immune B cells derived from immunized or disease convalescing subject can be immortalized by infecting with Epstein-Barr virus (EBV); then the virus transformed-immune B cells are cloned; individual clones grown* in vitro* similar to the mouse hybridoma culture and the secreted human monoclonal antibodies can be harvested from their culture supernatants [39–43]. Transgenic animals which their B cells carry human immunoglobulin gene loci produce human antibodies after antigen exposure [44–50]. Hybridomas secreting human monoclonal antibodies can be obtained from the immune transgenic animals [51]. Currently, several human monoclonal antibodies produced by transgenic animal lines have been approved for use in treatment of various human cancers and many more are in different stages of clinical development for various therapeutic purposes including allergy, autoimmune diseases, cancers, cardiovascular diseases, inflammatory diseases, infectious diseases, and pain [51, 52].

### 2.3. *In Vitro* Production of Human Single-Chain Antibodies by Using Phage Display Technology

Phage display technology invented by Smith in 1985 [53] has made* in vitro *production of human monoclonal antibodies specific to a desired target possible and relatively simple. Human immunoglobulin gene repertoire that resembles* in vivo* B lymphocyte pool (or even more diverse) can be generated* in vitro *by PCR amplification of human immunoglobulin gene amplicons derived from naïve, specifically immunized subjects, or synthetic gene pool and cloned into genome of a display system [54–56]. Degenerate primers can be used to obtain multiple amplicons from a single template [56]. Currently, many systems are available for the display purpose including yeast [57], bacteria such as* Escherichia coli *[58], mammalian cells [59], ribosomes [60], and phages (most commonly used) [56, 61].

**Figure 3:**
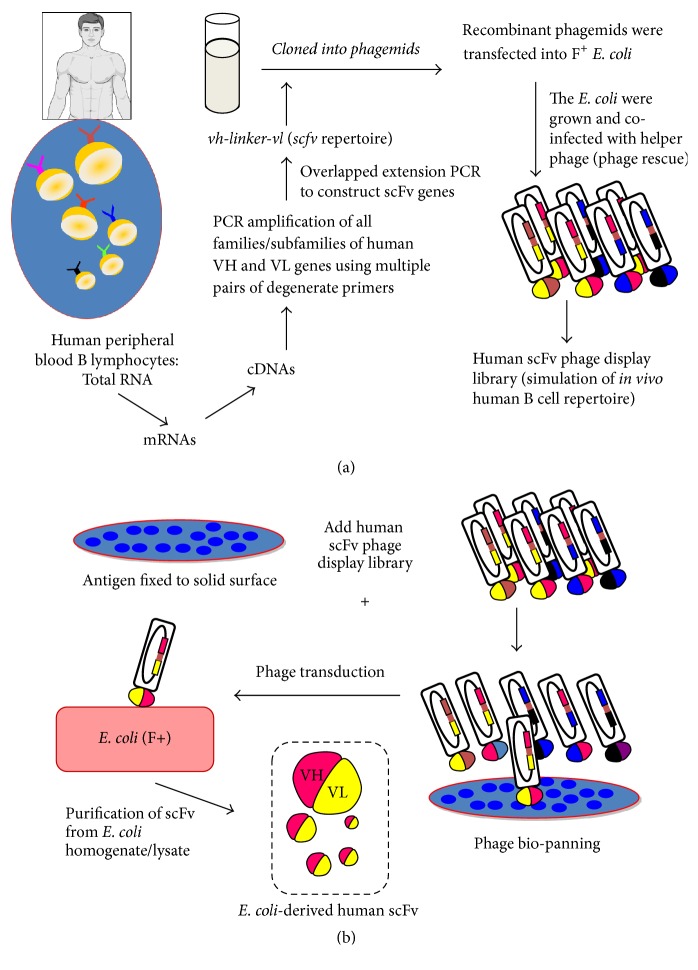
Procedures for (a) construction of human scFv phage display library and (b) phage biopanning and production of the* E. coli*-derived recombinant human scFv.

For constructing a human antibody-phage display library, genes of all human immunoglobulin families and subfamilies coding for diverse human antibody molecules (Fab, scFv, or sdAb) are PCR amplified and cloned into a phagemid vector (plasmid with a phage origin of replication) downstream of the phage gene coding for one of the coat proteins (pIII or pVIII) of the M13 phage [54, 56, 62]. An amber stop codon is inserted between the gene sequences of the antibody and the phage coat [62]. The recombinant phagemids are transformed into a special strain of* E. coli* that can produce tRNA of the stop codon (called suppressor* E. coli*). After growing and coinfecting the recombinant phagemid-transformed* E. coli* with a helper phage (such as M13KO7), the bacteria produce complete phage particles that individually display contiguous antibody-phage coat protein on their surface [62]. Each phage particle resembles a B lymphocyte, which contains antibody coding gene in the genome and concurrently displays the respective antibody on the surface as a fusion partner of one of the phage coat proteins. The antibody display phage library resembles a pool of B lymphocytes with diverse antigen-binding specificity. In our laboratory, a human scFv phage display library was constructed [56]. The overall process in constructing a human scFv phage display library and the production of bacterially derived human scFv is illustrated in Figures 3(a) and 3(b), respectively. Total RNAs were extracted from peripheral blood mononuclear cells of multiple blood donors and mRNAs were reversed-transcribed to cDNAs. Gene sequences coding for human VH and VL families and subfamilies were PCR amplified using the pooled cDNAs as templates and 14 forward and 3 reverse degenerate primers designed from multiple alignments of human functional immunoglobulin genes in the VBASE [63]. The amplified* vh* and* vl* sequences were linked together* via* a polynucleotide linker coding for a polypeptide composed of a triplicate of four glycines and one serine [(G_4_S)_3_] by means of spliced overlapped extension PCR (SOE-PCR) to generate DNA sequences* (huscfvs)* coding for human scFvs (HuscFvs) or VH-peptide linker-VL. The* huscfv* sequences were cloned into phagemid* via* the appropriate endonuclease restriction sites at the 5′ and 3′ ends, respectively. The recombinant phagemids were used to transform competent F+* E. coli* [the* E. coli* strain that a fertility factor (F) exists autonomously on the F episome (conjugative plasmid) in the cytoplasm; the bacteria express pili on their cellular surface which function in bacterial conjugation; the pili served also as receptors for filamentous phage transduction] by electroporation. The transformed* E. coli* were grown and cotransfected with helper phage and the complete phage particles were collected from the bacterial culture supernatant. The library has been used to generate human single-chain antibodies (HuscFvs) against several antigens including snake venoms, toxins, viral proteins, and other molecules as well as human proteins.

### 2.4. Single-Domain Antibodies (Nanobodies)

**Figure 4:**
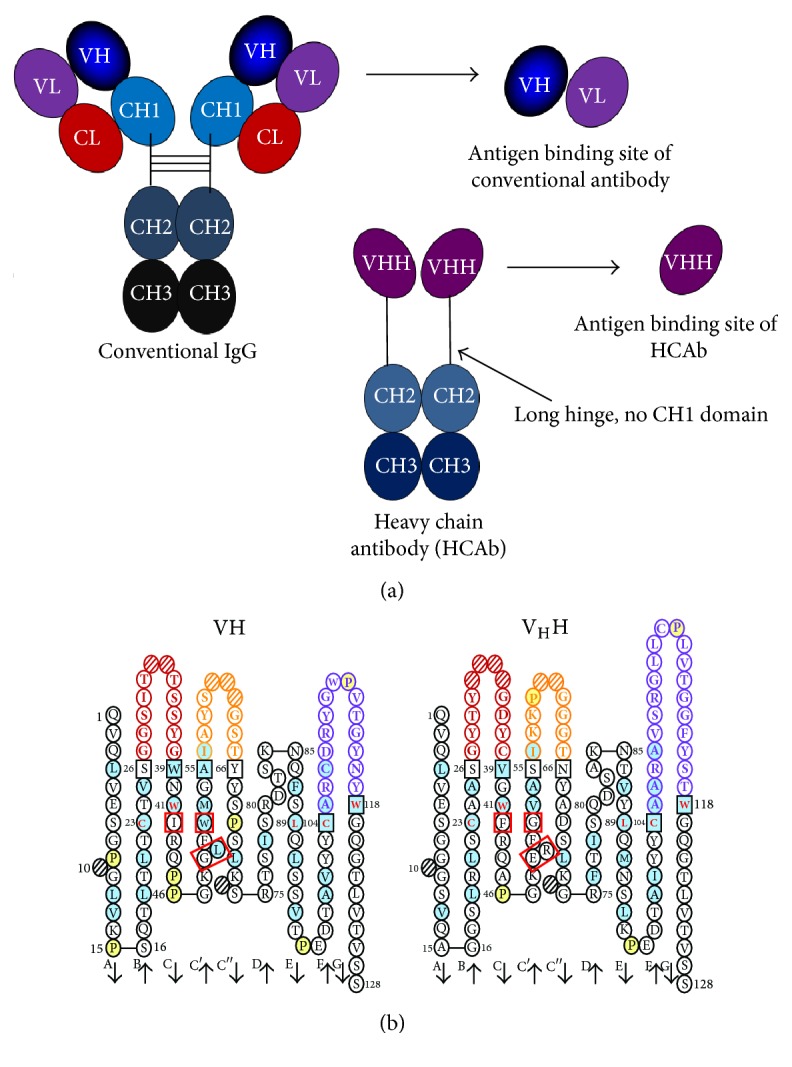
Diagrammatic representations of (a) conventional IgG, heavy chain antibody (HCAb), and antigen-binding sites of the conventional (VH and VL) and HCAb (VHH), respectively. (b) Deduced amino acid sequences of VH (left) and VHH (right). The immunoglobulin framework region-2 (FR2) of VH contains V/I 42, G49, L50, and W52 (red squares) while that of the VHH contains characteristic tetrad amino acids, that it, F/Y42, E49, R/C50, and G/L52 (red squares). The CDR3 of the VHH (purple circles of the right panel) is longer than that of the VH (purple circles of the left panel).

Based on differential adsorption on protein G- and protein A- affinity resins, serum of camelidae including one humped (old world) camels* (Camelus dromedarius*,* C. bactrianus), *llamas (*Lama glama* and* L. guanicoe*), and alpaca* (Vicugna pacos)* were found to contain three different IgG subclasses including IgG1, IgG2, and IgG3 [64]. The IgG1 which is conventional heterodimeric four-chain antibody with two heavy (H) and two light (L) polypeptide chains linked together by disulfide and noncovalent bonds (Figure 4(a), far left). The IgG2 (46 kDa) and IgG3 (43 kDa) are homodimers of H chains without the L chains; these dimeric H chain antibodies are called “Heavy chain antibody, HCAb” [64]. The two H chains of the HCAb are associated by noncovalent bonds. The H chain does not contain CH1 domain; the hinge region is relatively long compared to that of the conventional four-chain IgG. The antigen-binding site of the HCAb comprises only a variable domain of the H chain, designated variable domain of heavy chain of HCAb or VHH [65]. The VHH is linked to the hinge region followed by the Fc portion. Diagrammatic structure of the conventional IgG (IgG1 of camelids) and the antigen-binding site (VH-VL) in comparison to the HCAb structure and the HCAb antigen-binding site (VHH) are shown in Figure 4(a). Some hydrophobic amino acids in the region of the conventional VH that usually interacted with VL are mutated in the VHH to be more hydrophilic for reducing molecular aggregation [66]. This area is located on immunoglobulin framework region 2 (FR2) and contains characteristic tetrad amino acids including F/Y42, E49, R/C50, and G/L52 (substitute for V/I 42, G49, L50, and W52 of the conventional VH-FR2), according to IMGT numbering system [66] (Figure 4(b)). The tetrad amino acid hallmark is used for distinguishing the camel variable antigen-binding fragment (VHH) from the conventional VH. The sequence of the complementarity determining region 3 (CDR3) of the VHH is unusually long (16–18 amino acid residues) and longer than that of the human and mouse VH (average of 12 and 9 amino acid residues, resp.) [67]. The camelid VH and VHH can be engineered to express as single-domain antibody fragments (sdAb) of ~15–20 kDa which still retain antigen-binding capacity. The sdAb have about 10x lower molecular weight than the IgG and about 2x smaller than the scFv; therefore, they are called “nanobodies or minibodies” [68]. The genes coding for sdAbs can be cloned and expressed in* E. coli* system with relatively high yield, highly soluble in aqueous environments, and very robust [69, 70]. Because of their small sizes, the recombinant sdAbs are relatively stable to heat. They also have high binding affinity to the target [68, 69]. VHH antibodies have been shown to be potent enzyme inhibitors [71–75], as their long CDR3s can penetrate into the active pockets of the enzymes and block directly the respective catalytic activities which the conventional paratope consisting of VH and VL of the conventional four-chain antibody cannot do so [75, 76]. The sdAbs have become attractive therapeutic molecules for cancers, infectious diseases, parasitic infections, envenomation, intoxication, and inflammatory conditions caused by toxic enzymes [74, 77–87]. Camel V_H_H (Nanobody®, namely, ALX-0081, Ablynx, SOFINNOVA) specific to von Willebrand factor (anti-vWF) was tested in clinical trial in humans and found to be relatively safe without any untoward reactions in the recipients [87, 88]. Moreover, nanobodies have been used successfully as novel magic bullets for* in vitro *and* in vivo* immune-imaging for research and preclinical and clinical applications [89–92]. A phage library that displays humanized-camel VHs/VHHs was constructed in our laboratory [74]. The library was used subsequently in biopanning for selecting humanized-VHs/VHHs display phage clones that bound to a variety of targets including toxins [74, 82] and viral proteins [83–85].

**Figure 5:**
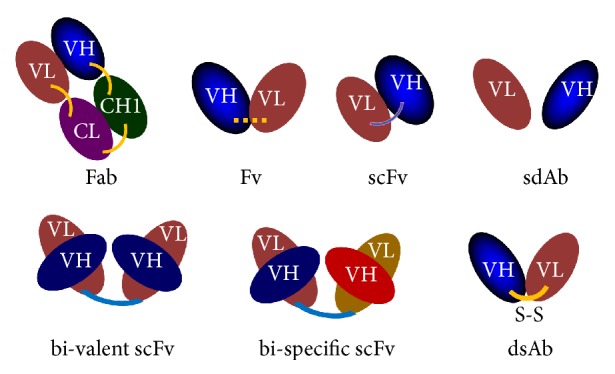
Some formats of engineered antibodies. FAb, fragment antigen binding [one light chain (VL and CL) is linked to VH and CH1 domain of heavy chain by disulfide bond]; Fv, variable fragments (VH and VL domains) are linked by chemical agent; scFv, single-chain antibody variable fragment where VH and VL domains are linked by a polypeptide; sdAb, single-domain antibody (VH or VL alone); dsAb, VH and VL domains are linked by disulfide bond.

Some formats of engineered antibodies are illustrated in Figure 5.

### 2.5. Cell Penetrating Antibodies (Transbodies)

Plasma membrane is a formidable barrier (because of the physicochemical properties) and only selectively allows permeability of certain small molecules (by means of passive diffusion, facilitated diffusion, or carrier proteins/transporters) such as gases, ions, water, sugars, amino acids, nucleosides, fat soluble vitamins [93]. A variety of biomolecules including antibodies are retained extracellularly. Antibodies are thus inaccessible to their intracellular targets such as proteins/enzymes of replicating virus, intracellular bacteria, or toxins that have entered the cells. To circumvent this obstacle, several delivery systems including cationic liposome [94], polyethyleneimine (PEI) [95], and peptides with cell penetrating capacity, called “Cell penetrating peptides, CPPs” [96, 97], have been developed for carrying antibodies/antibody fragments and a variety of other cargoes including proteins, drugs, nucleic acids, plasmids, and siRNAs across the plasma membrane into cytosol and also to different subcellular compartments [98–102]. CPPs have been used as a vehicle for cellular import of therapeutic molecules, both* in vitro* and* in vivo* [84, 85, 100–104]. Examples of CPPs are (1) protein transduction domains (PTDs) such as penetratin (PEN; synonym antennapedia homeodomain peptide of* Drosophila melanogaster*) [105], HIV-1 Tat peptide: Tat_49–57_ [106, 107], transportan (a 27 residue-peptide from galanin neuropeptide and mastoparan or wasp venom toxin) [97], and VP-22 peptide of structural protein of herpes simplex virus [108]; (2) amphipathic peptides such as noncytotoxic sweet arrow peptide (SAP) which is a proline-rich motif (VRLPPP) [105], peptide vector named MPG derived from the fusion sequence of HIV-1 gp41, and a hydrophilic domain of SV40 nuclear localization sequence [101]; and (3) other CPP type such as nonaarginine (R9) and poly-lysine [109]. In our laboratory, cell penetrable human scFvs and humanized-camel VHs/VHHs specific to viral proteins and toxins have been prepared by linking the antibody molecules to either penetratin or R9 [84, 85, 103, 104, 110–112]. These fusion proteins readily entered mammalian cells without causing cytotoxicity and bound to their respective intracellular targets. They were safe for mice after injecting repeatedly either intravenously or intraperitoneally at comparable doses to those given to humans for passive immunotherapy [111].

Comparison on some attributions of the conventional four-chain antibodies and engineered antibodies are given in Table 1.

## 3. Influenza and Influenza Viruses

### 3.1. Introduction

Influenza or flu caused by influenza viruses is a highly contagious respiratory disease of humans and animals worldwide [113]. The infection can lead to serious morbidity with high case-mortality rate especially among the elderly, small children, and individuals with chronic respiratory disease and/or immunocompromised condition. Influenza viruses are classified into four types, A, B, C, and D based on antigenicity of the viral nucleoprotein (NP) and major matrix protein (M1), epidemiologic patterns, host range, and symptom severity that they cause [114]. Only the type A viruses (IAV) have pandemic potential. In the human history, several catastrophic influenza A pandemics that led to many million deaths have been recorded periodically including Spanish influenza pandemic caused by IAV subtype H1N1 during 1918-1919 with estimated 20–50 million deaths [case-fatality rate (CFR) 2.3%], Asian flu caused by subtype H2N2 in 1957-1958 (~1–4 million mortality; CFR < 0.2%), and Hong Kong flu caused by H3N2 in 1968-1969 (~1–4 million deaths; CFR < 0.2%). In 1997, avian influenza H5N1 was transmitted from chicken to infect human and started the outbreak in Hong Kong which subsequently spread to other countries in Asia and other continents causing a high mortality rate, a global health threat, and a huge economic loss [115, 116]. In the first decade of this century, the World Health Organization (WHO) declared an influenza pandemic caused by a new H1N1 strain (subsequently named pandemic H1N1-2009, pdm09H1N1) as a Public Health Emergency of International Concern on April 26, 2009. This pandemic was started as an outbreak in Mexico where the infected subjects were succumb severe pneumonitis which led to high case-fatality ratio. The disease rapidly spread to the US and subsequently to 72 countries of other continents where hundreds of thousands subjects were infected and several hundreds were deceased [117]. Molecular analysis revealed that the virus contains genetic combination of H1N1 of the North American and the Eurasian swine lineages [118, 119]. Currently, different antigenically drifted strains of IAV subtypes H1N1 and H3N2 and type B (IBV) strains, either Yamagata or Victoria lineage, or both, are circulating and cause seasonal incidences of influenza annually [120, 121].

### 3.2. Biology of Influenza Viruses

Influenza viruses belong to family Orthomyxoviridae. They are enveloped negative-sense single stranded (ss) RNA viruses. Among the four types, the type A viruses (IAV) infect the widest host range including human, other mammals (both terrestrial and marine), bat, and avian species and inflict the most severe morbidity. They are divided further into subtypes according to the antigenic differences of two surface glycoproteins, that is, hemagglutinin (HA or H) and neuraminidase (NA or N). Currently, 18 HA and 11 NA subtypes have been recognized and the IAV virion may display any combination of the two proteins on the surface, for example, H1N1, H2N2, H3N2, H5N1 to H5N9, H7N1 to H7N9, H9N2, H10N7 [122]. Most of the IAV subtypes can be found in asymptomatic wild aquatic avian species except the two recently recognized subtypes, H17N10 and H18N11, which have been reported from bats [122, 123]. The B and C influenza viruses (IBV and ICV, resp.) infect principally humans. The former has similar host range and epidemic pattern to the IAV but never cause pandemic [124–127]. Since early 1980s, two lineages of IBV, Yamagata and Victoria, have been recognized [120]. Strains of the two lineages take preeminent of a particular year in causing human epidemic influenza B [128]. The C viruses (ICV) can cause swine infections that are transmissible to human and* vice versa* [127]. ICV infection may be asymptomatic or manifested only mildly [129]. Influenza D virus infects animals including cattle and pigs as well as humans (as shown by the presence of serum antibodies to the virus) but human case has not been reported [130–132].

**Figure 6:**
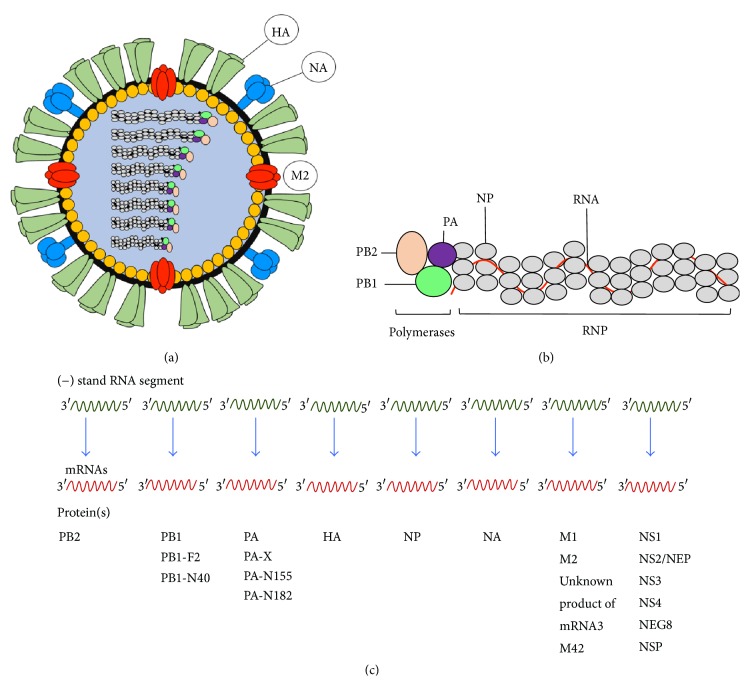
Influenza virus structure (a), genome segment organization (b), and gene products (c).

Influenza virus structure, genome segment organization, and gene products are shown in Figure 6. Bioactivities of the influenza viral proteins are detailed in Table 2. Genomes of IAV and IBV contain 8 RNA segments that code for 18 functionally different proteins [133], while the ICV and IDV have 7 gene segments as they lack the fifth segment coding for neuraminidase. The first gene segment of influenza virus encodes basic polymerase-2 (PB2) which is translated from AUG1 of the nonspliced mRNA [133]. The second RNA encodes another basic polymerase, PB1, and two other proteins, that is, PB1-F2 and PB1-N40 [133]. Products of the third RNA segment are acidic polymerase (PA), PA-X, PA-N155, and PA-N182 proteins [133]. The fourth, fifth, and sixth RNA segments code for HA, NA, and NP, respectively. The seventh gene encodes major matrix protein (M1), ion-channel protein (M2), and M42. Products of the last gene include NS1, NS2, or nuclear export protein (NEP), NS3, NS4, NEG8, and NSP, respectively [133]. Each gene segment of the influenza virus is encased by NP and binds one molecule each of PB1, PB2, and PA to form RNA-dependent RNA polymerase (RdRp) complex for viral transcription and replication [134]. The PB1 has endonuclease activity that can excise cap structure (G^7m^) from the host pre-mRNA for initiation of the viral transcription [135–137]. This protein also contains RNA-dependent RNA polymerase (RdRp) motif and binds to vRNA and cRNA promoters [138]. The PB2 recognizes and binds to the cap that the PB1 snatched from the host pre-mRNAs [114, 139]. PA is involved in the viral transcription and replication [140–143]. The PB1-F2 translated from an alternate reading frame (AUG4) of the PB1 mRNA [144] has been shown to impair the cellular innate immunity by accelerating mitochondrial fragmentation [144]. PB1-N40 is another polypeptide of the second gene segment that is translated from AUG5 due to leaky ribosomal scanning [133]. This protein is an N-terminally 39 residue-truncated PB1 whose function is to maintain the balance of expressions of the PB1 and the PB1-F2 [133, 145]. PA-X (292 residues) is a PA overlapping protein that the first 191 N-terminal amino acids are identical to PA but the PA-X possesses 61 specific amino acids at its C terminus [146, 147]. However, most human pdm09H1N1 and swine viruses possess only 41 PA-X-specific amino acids [138, 147]. The PA-X is translated from AUG1 and +1 ribosomal frame shifting (codons 190–193) of the third gene segment [133, 146, 148]. This protein possesses endonuclease activity [149] and contributes to viral growth and virulence and host immune response suppression [133, 149]. PA-N155 (translated from nonspliced mRNA of gene segment 3 at AUG11 due to leaky ribosomal scanning) and PA-N182 (translated from AUG13 of the third gene segment) are N-terminally truncated forms of PA which does not have polymerase activity [133, 150]. The proteins of PA gene could be detected in infected cells of many host species and may be involved in influenza pathogenicity [150]. Virus mutants lacking the proteins replicated slowly in cell culture and conferred low pathogenicity in the infecting mice [149]. Hemagglutinin (HA) is the product of the fourth influenza gene segment. It is one of the two glycoproteins that decorate the influenza virus envelope. The protein is produced as a trimeric 76 kDa HA0 molecule from endoplasmic reticulum of the infected cell and is transported through Golgi apparatus to the plasma membrane [151, 152]. The HA0 must be nicked posttranslationally into two disulfide-linked HA1 (~58 kDa) and HA2 or hemagglutinin stalk (~26 kDa) to become a functional HA molecule [153, 154]. This viral protein is the principal target of vaccine-induced neutralizing antibodies which provide protective immunity against influenza. HA has a major role in the early stage of infection including host receptor binding for virus entry (function of the HA1 domain) and viral-endosomal membrane fusion for cytoplasmic entering of the vRNPs (activity of the HA2) [155]. For the cellular entry, HA uses HA1 domain to interact with host cell receptors which comprises terminal sialic acid linked to galactose residue* via* either *α*2,3 or *α*2,6 linkage [155]. In the acidic endosomal environment, the endocytosed vRNPs are released from the M1 protein, while the HA molecule undergoes conformational change to expose HA2 peptide that causes fusion of the host and the viral membranes and an exit of the vRNPs into cytoplasm for further transportation to nucleus where the viral RNA replication takes place [156]. Neuraminidase (NA) (~65 kDa) is the only product of the sixth gene segment. NA is another surface glycoprotein of the influenza viral particle. This viral enzyme digests the sialic acid receptors on the host cell to free the newly formed virus particles for further spread [157]. NA has been shown to be important for initiating influenza virus infection in human airway epithelium by cleaving sialic acid in the extracellular matrix to facilitate the HA binding to the cellular receptors [158]. NA also limits viral superinfection (infection of a target cell by more than one virion) of the infected cells [159]. The seventh RNA segment of influenza A viruses produces 4 mRNAs [160]. The mRNA1 is translated into major matrix protein (M1) which is the product of the unspliced mRNA transcript [133]. M1 mediates self-oligomerization to form meshwork that lies underneath the influenza viral envelope for maintaining the virion integrity [161, 162]. The M1 matrices are important for assembly of newly synthesized viral components and budding of the progeny viruses [163–168]. At the early stage of infection, M1 frees endosomal vRNP into cytoplasm for further replication in the nucleus [168]. M1 which contains nuclear localization sequences (NLSs) interacts with C-terminal domain of the nuclear export protein (NEP) (which also has NLSs) and NP for transporting newly synthesized vRNPs from nucleus to cytoplasm for further assembly and budding [169–172]. M1 also prevents the newly formed vRNPs from reentering the nucleus [173]. M2 of influenza virus is the second product of the seventh RNA segment. The protein is translated from the spliced transcript (mRNA2) of the gene [160]. It is a type III membrane protein of the viroporin family [174]. This protein forms homotetramers on the membrane of the virus infected cells and, to a fewer extent, on the virion surface. M2 functions as a pH-activated ion channel which allows H^+^ to enter the virion causing a release of vRNP into cytoplasm for further replication in the nucleus [175]. M2 interferes with cellular macroautophagy by stimulating autophagosome formation during the early phase of the infection but blocks fusion of the autophagosomes to lysosomes at the late phase which consequently compromises survival of the infected cells for viral fitness [176]. The protein prevents acid-induced conformational change of newly produced HA in trans-Golgi network [177]. At the late stage of the viral infection, M2 is recruited by the M1 to the site of virus budding where the amphipathic helices of the M2 cause plasma membrane curvature and membrane scission for virion release [178]. Protein product of the mRNA3 of the seventh gene segment is not known. M42 is a newly recognized protein of a variety of IAV which is translated from a spliced variant mRNA4 of the seventh gene segment [179]. The M42 has distinct ectodomain from the M2 and can replace the M2 functions in the M2-deprived viruses [179]. The eighth gene segment of influenza virus encodes several proteins, also by differential RNA splicing [160]. The first product of the 8th gene segment is NS1 which is a multifunctional and relatively conserved protein of influenza viruses [180]. NS1 is translated from nonspliced mRNA (AUG1) [181]. At the early phase of the viral infection, the protein suppresses host immunity by competing with cytoplasmic and endosomal pattern recognition receptors (PRR) for binding to viral RNA and thus interferes with the host innate interferon (IFN*α*/*β*) signaling and production. Inhibition of the innate interferon production consequently inhibits generation of several antiviral factors such as 2′,5′-oligoadenylate synthetase (OAS) and protein kinase R (PKR) and prevention of cellular apoptosis [182–187]. NS1 inhibits host protein synthesis and enhances viral translation [188–192]. The protein interferes with maturation and migration of dendritic cells (DCs); thus, the cells were unable to stimulate immune responses, especially cell-mediated responses [193]. NS2/NEP is translated from alternatively spliced mRNA, NS2 mRNA (also AUG1) of the 8th gene segment [133]. C-terminal domain of the NS2/NEP binds vRNP-bound-M1 while the N-terminal domain interacts with nuclear export protein, named chromosome region maintenance 1 (CRM1), and mediates nuclear export of the newly synthesized vRNPs [170, 194]. The NS2/NEP is critical for the influenza virus replication cycle [195]. NS3 protein is a novel protein of the 8th gene segment of influenza virus that mutated from the NS1 by 374A→G substitution encoding D125G during improvement of the human virus adaptation to mice [196]. This protein has similar sequence to the NS1 but with internal deletion of a motif coded by codons 126 to 168 [196]. The 8th RNA segment of human, swine, and avian influenza viruses may contain alternative open reading frames (ORFs) that encode additional polypeptides besides the NS1, NEP, and NS3 including NS4 [133], NEG8 (translated from either 167 or 216 codon on genomic/negative RNA strand) [197, 198], and a 19 kDa negative-strand protein (NSP) where the ORF is located in positive sense orientation in the negative-sense 8th RNA segment [199].

### 3.3. Influenza Clinical Features

Human gets influenza through different modes of viral transmission including direct contact with the infected subjects, inhalation of the virus-laden respiratory droplets from the infected subject released out by coughing, sneezing, and/or talking, or by fomites (contact with influenza virus contaminated objects and transmit the virus to oral/nasal mucosa) [200]. Influenza viruses in the nasopharynx are usually trapped by the mucus which contains sialic acid-linked glycoproteins. Neuraminidase (NA) plays role in facilitating the viral entry to the epithelial cells lying underneath the mucus by cleaving the sialic acids from the mucus glycoproteins to free the mucus trapped-virus [201]. HA attachment to the receptor allows cellular entry of the virus. Symptoms of influenza usually appear after a period of about 24–48 hours. The clinical signs include chill, fever, malaise, myalgia, fatigue, headache, sore throat, cough, and stuffy or running nose. Not all patients with flu have all of the clinical manifestations [202]. Some patients may have also diarrhea and vomiting. The flu symptoms tend to last for several days but usually subside within two weeks. However, small children, elderly (65 years or older), pregnant women, and people with underlying chronic diseases such as allergic asthma and chronic lung disease are at risk to severe complications including primary viral pneumonia (uncommon) [201] and secondary pneumonia caused by bacteria such as* Staphylococcus aureus, Haemophilus influenzae, *and* Streptococcus pneumoniae* [203].

### 3.4. Treatment of Influenza by Using Pharmacologic Drugs

There have been only two families of FDA-approved direct acting drugs for influenza treatment [204–206]. The first drug family is blockers of polymeric M2-mediated ion-channel activity. These drugs are derivatives of adamantanes for oral administration including rimantadine and amantadine. The M2 blockers are the first generation anti-influenza drugs that aimed to prevent the endosomal exit of the vRNP into cytoplasm. Another group of anti-influenza drugs are neuraminidase inhibitors (NAIs), including oral oseltamivir phosphate (which is commercially available under the name Tamiflu), inhaled zanamivir (commercially available as Relenza), laninamivir octanoate or CS-8958 (long-acting inhaled NAI) (commercially available as Inavir), and parenteral peramivir for treatment of patients with IAV pdm09H1N1 infection [207]. The NAIs inhibit the release of the virus progeny from the infected cells and the viral spread [205, 206]. For high therapeutic effectiveness, the anti-influenza drugs should be taken at the early phase of the infection [206]. Currently, however, influenza viruses of most, if not all, IAV subtypes as well as IBV become resistant to the ion-channel blockers mainly by M2 mutations [208] leading to treatment failure [209, 210]. Resistance to oseltamivir, zanamivir, and peramivir has emerged [211–213]. This is by mutations in the NA molecule that cause alteration of the shape of the NA catalytic site [207]. The anti-influenza drugs also have toxicity and side effects [205]. Besides, their supply may not be able to cope with a high demand at the disease upsurge. Sought of novel anti-influenza agents that are safe and effective against multiple strains with high tolerability to the viral mutations (antigenic drift or shift) is an active area of investigation. These may be either substances that target virus proteins or genes or molecules that inhibit host factors essential for the viral replication [214]. Experimental compounds/substances and their molecular mechanisms for future influenza treatment and management have been reviewed elsewhere [214, 215].

### 3.5. Active Immunization against Influenza

Annual vaccination against influenza is recommended for general population older than 6 months [206]. Seasonal influenza vaccines (flu shots) may be trivalent (consisting of three strains, one strain each of H1N1 and H3N2 and one IBV strain, usually Yamagata lineage) or quadrivalent (four influenza strains, one strain each of IAV H1N1 and H3N2 and one strain each of IBV Yamagata and Victoria lineages) [120]. Virus strains in the vaccines must be changed annually as the immunity induced by the vaccines in the previous year confers inadequate, if any, protection against the viruses of the following years due to rapid antigenic drift of the circulating viruses. Usually the World Health Organization (WHO) recommends the viruses that are antigenically and genetically matched with the causative viruses of the particular flu season for influenza vaccine inclusion. For this instance, the WHO obtained the antigenic and genetic data of the causative viruses of a particular flu season from the WHO Collaborating Centers of the Global Influenza Surveillance and Response System (GISRS). The recommended candidate vaccine strains are different also for northern and southern hemisphere influenza seasons. Although the vaccination is the most effective measure for intervention of the influenza virus infection and severe clinical manifestations caused by IAV and IBV [216], there are hurdles in production, use, and efficacy of the seasonal influenza vaccines [217]. Influenza vaccine viruses are propagated mostly in specific pathogen-free 8–10-day-old embryonated eggs. Thus, not only the vaccines are contraindicated for the egg-allergic subjects, but also the supply is limited at a high demand such as during large epidemics. The vaccines also contain some ingredients that might cause allergic reaction in the vaccinated subjects [218, 219]. Besides, small children, elderly, people with certain medical conditions and immunocompromised individuals which are the risk group to severe influenza complications may not be well protected by the influenza vaccination [220]. New generation egg-independent vaccines such as animal cell culture-based and DNA vaccines that induce broadly protective immunity against influenza are required [221, 222]. There is no vaccine for ICV infection.

## 4. Immunotherapy of Influenza and Perspective

Antibodies have been used with high success in influenza therapy [223–230]. Currently, investigations on human monoclonal antibodies for passive immunization against influenza have been a focus of intensive research. The effective monoclonal antibodies should be important for both pre- and postviral exposure for intervention of morbidity or reduction of symptom severity as well as for treatment of severe influenza, especially in the immunocompromised subjects or infections with drug resistant viruses [18, 230]. Moreover, it has been mentioned [18, 230] that it is feasible that passive immunization with broadly effective antibodies implemented early in an unprecedented/unpredicted influenza pandemic should mitigate the health and economic impact caused by the newly emerged virus when the vaccine against the pandemic strain is not ready for wide distribution. The monoclonal antibody products that are under clinical development, challenges, and potential applications for passive immunization against influenza have been reviewed elsewhere [231, 232]. It is known that frequent mutations of the surface-exposed proteins, particularly receptor binding domain of influenza virus hemagglutinin, lead to reduction or abrogation of affinity and efficacy of anti-hemagglutinin antibody which is the principal protective antibody against influenza. Therefore, the vaccine for active immunization as well as the antibodies for passive immunization should target the highly conserved epitopes of the virus proteins. Existing data indicated that antibodies that bind to a conserved region of the influenza virus surface-exposed proteins such as membrane-proximal portion of hemagglutinin stalk or ectodomain of M2 (anti-M2e, TCN-032) conferred broad protection against various subtypes of IAV both* in vitro *and* in vivo* challenges in mice, ferrets, and humans [232–236]. Nevertheless, there has been an immunological concern that the intact antibody molecule might mediate antibody-dependent enhancement (ADE) of influenza virus infectivity which potentially leads to aggravation of symptom severity if used in treatment of patients with severe influenza [231, 237–239]. Increased infection rates of pandemic influenza 2009 H1N1 have been found following seasonal flu vaccination [240, 241]. Severely ill patients that were infected with the pandemic H1N1 had concomitant immune complexes of preexisting low avidity and nonprotective antibodies from previous exposure to seasonal circulating influenza viruses and fatal cases had complement product, C4d, in the lung [242]. Piglets immunized with inactivated H1N2 virus had enhanced severity upon challenged with pandemic H1N1 virus due to predominant nonprotective anti-HA stalk antibody [239].

The strategy that we are proposing for passive immunization against influenza is the use of fully human single-chain antibodies (HuscFvs) that target conserved regions of pivotal proteins of the influenza viruses including surface-exposed, secreted, and internal proteins. The fully human single-chain antibodies should be safe as they devoid of the Fc portion; thus, they cannot cause ADE in the treated subjects. Human scFv phage display library constructed in our laboratory [56] has been used as a biological tool for providing HuscFv display phage clones that bound to the desired influenza virus targets. Recombinant influenza virus proteins with the inherent functional activities or intact virus adsorbed to cell surface were used as antigens in the phage biopanning process [103, 104, 243–245]. The antigen bound phages were then put in nonsuppressor* E. coli *that could not produce tRNA for the stop codon located between the antibody coding gene* (huscfv)* and the phage p3 gene. These phage-transformed* E. coli *were grown in appropriate medium to express soluble HuscFvs. The HuscFvs produced by individual phage transformed* E. coli* clones were tested for specific binding to the targets by appropriate immunoassays. DNA sequences coding for the HuscFvs of interest and their complementarity determining regions (CDRs) and immunoglobulin framework regions (FRs) were determined. Computerized simulation by means of homology modeling and intermolecular docking between the antibodies and the modeled or existing three-dimensional (3D) structures of the targets were used for guiding selection of the clones that produced target-bound HuscFvs. For large scale production of the HuscFvs, the* HuscFv* sequence in the phagemid was subcloned into protein expression plasmids containing strong promoters, such as that of bacteriophage T7. The recombinant plasmids were then introduced into* E. coli* expression host. These* E. coli *usually produce relatively high amounts of the HuscFvs as inclusion bodies (IBs). The IBs can be isolated from the bacterial homogenates and washed extensively to eliminate as much as possible the host contaminants; then the HuscFvs in the IBs were refolded [111]. The refolded antibodies were retested for target binding and affinity. Functional inhibition assays of the target molecules are then performed before testing the fully human antibodies further for protective activities.

Human scFvs that neutralize receptor binding activity of homologous and heterologous strains and clades of highly pathogenic H5N1 were generated from phage clone derived from panning with recombinant H5 of clade 1 virus [243]. Therapeutic efficacies of the HuscFvs were tested in a mouse model of influenza [243]. HuscFvs from one of the* E. coli* clones readily rescued C57BL/6 mice from lethal challenge with heterologous H5N1 [243]. HuscFvs that bound to A/H5N1 M2 residues important for ion-channel activity, macroautophagy, M1 binding, and amphipathic helices-mediated viral budding and release were produced [244]. The antibodies inhibited replication of heterologous influenza viruses in the infected cell cultures [243] and protected infected mice from lethal influenza (unpublished data). Because NS1 is a multifunctional virulent factor that is indispensable of the influenza virus replication cycle, we produced HuscFvs that bound to both recombinant and native influenza virus NS1 [245]. HuscFvs of one phage-transformed* E. coli *clone reacted with NS1 RNA binding (R) domain important for host innate immune response suppression. HuscFvs of two other clones bound to different sites of the NS1 effector (E) domain; one of them docked on the NS1 site important for host elF4G1 protein binding while another interacted with residues of NS1 that usually bind to host CPSF30 protein for intervening host 3′ end pre-mRNA processing [245]. These HuscFvs not only inhibited replication of influenza viruses across types and subtypes (H5N1 strains isolated from duck and dog, swine H1N1, seasonal human H1N1 isolated in Thailand, California H1N1, swine H3N2, Perth H3N2, and Brisbane B strain), but also could restore the host innate immune response by upregulating the* IRF3* and* IFN-β* genes that had been suppressed by the infecting influenza viruses [245]. Influenza virus M1 protein has several pivotal roles in the influenza infectious cycle as mentioned above; thus this protein is another attractive target of anti-influenza agent. Each molecule of M1 contains 252 amino acids which can be separated into N-terminal domain (ND; residues 2–67), middle domain (MD; residues 91–158), and C-terminal domain (CD) [246, 247]. HuscFvs specific to M1 were produced by using the human scFv phage display library as the source of antibody genes [103]. HuscFv from one phage transformed* E. coli* clone bound to recombinant and native M1 of various A virus subtypes including highly pathogenic avian influenza (HPAI) H5N1, H1N1, and H3N2, as well as the less pathogenic H8N4 [103]. The antibody blocked native M1 binding to RNA [103]. The HuscFv was developed into a cell penetrable format (transbody) by linking the antibody molecule to a cell penetrating peptide (CPP) [103]. The CPP-HuscFv readily traversed across the plasma membrane and bound to the M1 of the replicating virus in the infected cells without causing any significant cytotoxicity [103]. The transbody reduced the amounts of virus released from the infected cells compared to the infected nontreated cells [103]. Because the middle domain of the M1 contains regions that mediate multiple pivotal functions of the influenza virus replication cycle, that is, nuclear localization signal (NLS), NEP binding motif, RNA/RNP-binding site, transcription inhibition motif, self-oligomerization domain, and plasma membrane interactive site for assembly and budding [161, 165, 166, 170, 246–251], we also produced cell penetrable HuscFv (transbody) to the M1-MD [104]. This transbody extricated mice from lethal infection with mouse adapted HPAI H5N1 by mitigating symptom severity and reducing lung histopathology of the treated infected mice. We envision that the fully human single-chain antibodies specific to the influenza virus proteins have high potential for testing further as another ramification of influenza therapeutic agents.

## 5. Conclusions

By means of contemporary technologies, antibodies that are fully biocompatible to the human immune system (no immunogenicity in the recipients) for a safe passive immunization against infections and noninfectious diseases can be generated* in vitro* without a prolonged* in vivo* immunization process. In our laboratory, human single-chain antibodies to different pivotal proteins of influenza virus that could rescue infected mice from lethal challenge with influenza viruses of different subtypes were produced. The antibodies are small and devoid of Fc portion; hence, they should have high tissue penetrating ability and cannot cause ADE, respectively. For targeting intracellular virus proteins, the antibody fragments can be made into a cell penetrable format (transbodies) by linking them molecularly to a nonimmunogenic, noncytotoxic cell penetrating peptide (CPP), such as nonaarginine (R9) [111, 252]. The cell penetrating antibody, term superantibody (coined by Köhler and Paul in 1998 [253]) can cross the membrane of all cells but get accumulated intracellularly only where the target antigen is present. For passive immunization against influenza which is an acute infection, the ready-made antibody can be injected daily for 3-4 doses to mitigate the disease severity [242]; therefore,* in vivo* longevity of the antibody fragments should be of less concern than for treatment of chronic diseases, such as cancers, HIV infection, or viral hepatitis. The potential limitations and challenges during the development of these engineered antibodies towards the therapeutic purpose, such as up-scaling and proper refolding ability of the* E. coli*-derived-HuscFvs, should be solved by changing the expression host system to mammalian cells such as Chinese hamster ovarian (CHO) cells which are the World Health Organization-approved cells for large scale production of therapeutic proteins [254]. The recombinant small antibody fragments to influenza proteins await preclinical and clinical trials towards the application as a novel, broadly effective, and safe anti-influenza agent.

## Figures and Tables

**Table 1:** Comparison on some attributions of the conventional four-chain antibodies and engineered antibodies.

Attribution	Conventional four-chain antibodies	Engineered antibodies (four-chain and fragments)
Selection	*In vivo* or achieved *via* hybridoma technology which need repeated animal immunization Hybridoma technology requires tissue culture facility and hand on experience Antigen: must be immunogenic with appropriate dose, route, etc. Host factors to be considered: genetics, MHC and immune status	*In vitro*: antibody coding genes can be selected from bacteria, yeast, and mammalian display systems Animal-free system (alleviates animal welfare concern). Antigen: no restriction Free from influence of host status

Generating time	Relatively long process	Relatively short-time (less than 4 weeks to get antigen binding clones from the display systems)

Production	Hybrodomas require tissue culture facility and expensive culture medium	Various and flexible expression systems including bacterial, yeast, and mammalian High yield can be obtained, for example, from optimal mammalian expression system

Reproducibility	*In vivo*: animal-to-animal and batch-to-batch variation in quality	Low batch-to-batch variation No life-time limit

Genetic stability	Genetic drift (hybridomas)	Relatively more stable

Molecular structure	Mostly unknown	Known DNA sequence information, defined structure (CDRs and FRs)

Format	Four chains with strict species, isotypes, subisotypes	Can be four-chain or engineered at genetic level to preferable formats (to suit the purpose of use): chimeric, humanized, fully human, F(ab)′2, Fab, scFv, sdAb, multi-valent, multimeric, and many other possibility

Purity	Antibodies from *in vivo* immunization and hybridoma culture can be contaminated with the host proteins, and disease causative and adventitious agents from animal derived raw material	Can be purified to be free from adventitious agents with high purity (up to 99.8% at GMP level) Animal-free raw material

Affinity	Usually high but cannot be improved or modulated	Can be improved and modulated by *in vitro* affinity maturation, point mutation(s) or resurfacing of the antigen binding site

Cell penetrating ability	No; inaccessible to intracellular target	Yes, by linking molecularly to a cell penetrating peptide; thus, can be accessible to the intracellular target

Half-life *in vivo*	Can be several weeks (isotype-matched)	Can be many hours to several weeks depending on the designed format; increased longevity and pharmacokinetics can be done, such as by PEGylation, multimerization, or modulating IgG/FcRn interaction The cell-penetrating antibody (superantibody) can stay *in vivo *for relatively long period of time as they can cross the membrane of all cells but get accumulated intracellularly only where the target antigen is present. Thus, disappearance of the superantibodies from the blood circulation does not imply that they were eliminated from the body

Fc fragment	The antibody has functional capabilities that are mediated by the Fc including complement dependent cytotoxicity (CDC), antibody-dependent cell-mediated cytotoxicity (ACDD), opsonization, and immune complex removal; nevertheless, the Fc function is derived by chance	Antibody fragments devoid of Fc usually do not cause Fc-mediated inflammation. They cannot mediate CDC, ADCC, opsonization and immune complex removal. Engineered four-chain antibodies can be designed for appropriate immunological functions such that their Fc can fix properly to receptors, either activating receptors, such as Fc*γ*RI, Fc*γ*RIIa, Fc*γ*RIIIa (CD16) or inhibitory receptor (Fc*γ*IIb)

Tissue penetrating ability	Relatively low, mostly depends on their interaction to FcRs Tend to comigrate with FcR-bearing immune cells	Relatively high, due to small size and no Fc restriction; they can freely migrate to the site of infection/affected areas (high tissue penetration)

Antibody-dependent enhancement (ADE) of viral infection	Frequent for many viral infections, such as Dengue, influenza, Zika, Chikungunya, West Nile, and HIV-1	Relatively safe for use in treatment of various viral infections as the antibody fragments devoid of Fc do not have ADE ability, while Fc fragments of intact four-chain engineered antibodies can be modified to abrogate Fc receptor binding ability

Side effects	Uncontrolled binding site, affinity, and Fc function Can cause adverse effects such as serum sickness, tumor lysis syndrome, cytokine release syndrome, and anaphylaxis	Minimized potential for causing adverse effects can be achieved through modulation of binding site and affinity, humanization, and Fc engineering

**Table 2:** Functions of influenza virus proteins.

Gene segment	Name of protein	Function(s)
1	PB2	Basic polymerase recognizes and binds to the cap that the PB1 snatched from the host pre-mRNAs for genome replication

2	PB1	Basic polymerase with endonuclease activity that can excise cap structure (G^7m^) from the host pre-mRNA for initiation of the viral transcription
	PB1-F2	Impair the cellular innate immunity by accelerating mitochondrial fragmentation
	PB1-N40	Maintains the balance of expressions of the PB1 and the PB1-F2

3	PA	Acidic polymerase which involves in the viral transcription and replication
	PA-X	Possesses endonuclease activity and contributes to viral growth and virulence and host immune response suppression
	PA-N155 and PA-N182	Do not have polymerase activity; likely possess important functions in the replication cycle of influenza A virus as virus mutants lacking these proteins replicate more slowly in cell culture and have lower pathogenicity in mice

4	HA	Plays major role in the early stage of infection by binding with host cell receptors for virus entry (function of the HA1 domain) and viral-endosomal membrane fusion for cytoplasmic entering of the vRNPs (activity of the HA2) for further virus replication in nucleus

5	NA	Digests the sialic acid receptors on the host cell to free the newly formed virus particles for further spread Cleaves sialic acid in the extracellular matrix to facilitate the HA binding to the cellular receptors at the initial stage of infection NA also limits viral superinfection of the infected cells

6	NP	NP encapsidates the viral RNA and binds one molecule each of PB2, PB1, and PA to form RNA-dependent RNA polymerase (RdRp) complex for viral transcription and replication Later in the infection, NP is bound by M1 which mediates nuclear export of newly produced vRNPs through interacting with nuclear export protein, NEP It is also possible that NP binds directly host exportin-1/XPO1 and plays an active role in RNPs nuclear export

7	M	M1: at early stage of infection, M1 releases endocytosed vRNP while the HA molecule undergoes conformational change to expose HA2 peptide that causes host-viral membranes fusion and an exit of the vRNP into cytoplasm for further transport to nucleus where the viral RNA replication takes place M1 interacts with NEP and NP for transporting newly synthesized vRNPs from nucleus to cytoplasm for further assembly and budding M1 also prevents the newly formed vRNPs from re-entering the nucleus M2 forms ion channel at the viral membrane which allows H^+^ to enter the virion causing vRNP release into cytoplasm for further replication in the nucleus M2 initiates autophagosome formation at the early stage of infection but blocks autophagosome fusion to lysosome at the late phase which consequently compromises survival of the infected cells for viral fitness M2 prevents acid-induced conformational change of newly produced HA in trans-Golgi network At the late stage of the viral infection, M2 is recruited by M1 to virus budding site where the M2 amphipathic helices cause plasma membrane curvature and membrane scission for virion release M42 can replace the M2 functions in the M2-deprived viruses

8	NS	NS1 suppresses host immunity, inhibits host protein synthesis and enhances viral translation NEP mediates nuclear export of the newly synthesized vRNPs NS3 might involve switching from avian to mammalian hosts, including human, swine and canine populations NS4, NEG8, and NSP: unknown functions

## References

[1] Casadevall A., Pirofski L. A. (2004). New concepts in antibody-mediated immunity. *Infection and Immunity*.

[2] Casadevall A., Pirofski L. A. (2012). A new synthesis for antibody-mediated immunity. *Nature Immunology*.

[3] Wesselhoeft C. (1936). Treatment of scarlet fever and diphtheria. *Medical Clinics of North America Journal*.

[4] Keller M. A., Stiehm E. R. (2000). Passive immunity in prevention and treatment of infectious diseases. *Clinical Microbiology Reviews*.

[5] Gonik B. (2011). Passive immunization: the forgotten arm of immunologically based strategies for disease containment. *American Journal of Obstetrics & Gynecology*.

[6] Chames P., van Regenmortel M., Weiss E., Baty D. (2009). Therapeutic antibodies: successes, limitations and hopes for the future. *British Journal of Pharmacology*.

[7] Li J., Zhu Z. (2010). Research and development of next generation of antibody-based therapeutics. *Acta Pharmacologica Sinica*.

[8] Sathyanarayanan V., Neelapu S. S. (2015). Cancer immunotherapy: strategies for personalization and combinatorial approaches. *Molecular Oncology*.

[9] Khardori N. (2006). *Bioterrorism Preparedness*.

[10] Casadevall A. (1996). Antibody-based therapies for emerging infectious diseases. *Emerging Infectious Diseases*.

[11] Prabhakar D. R., Motiram M. V., Ghanshyam B. C. (2014). Antivenoms in snake envenoming: are they safe?. *Journal of Clinical Toxicology*.

[12] Sapsutthipas S., Leong P. K., Akesowan S., Pratanaphon R., Tan N. H., Ratanabanangkoon K. (2015). Effective equine immunization protocol for production of potent poly-specific antisera against *Calloselasma rhodostoma*, *Cryptelytrops albolabris* and *Daboia siamensis*. *PLOS Neglected Tropical Diseases*.

[13] Köhler G., Milstein C. (1975). Continuous cultures of fused cells secreting antibody of predefined specificity. *Nature*.

[14] Chaicumpa W., Thin-Inta W., Khusmith S. (1988). Detection with monoclonal antibody of *Salmonella typhi* antigen 9 in specimens from patients. *Journal of Clinical Microbiology*.

[15] Chaicumpa W., Srimanote P., Sakolvaree Y. (1998). Rapid diagnosis of cholera caused by *Vibrio cholerae* O139. *Journal of Clinical Microbiology*.

[16] Saengjaruk P., Chaicumpa W., Watt G. (2002). Diagnosis of human leptospirosis by monoclonal antibody-based antigen detection in urine. *Journal of Clinical Microbiology*.

[17] Mease R. C., Foss C. A., Pomper M. G. (2013). PET imaging in prostate cancer: focus on prostate-specific membrane antigen. *Current Topics in Medicinal Chemistry*.

[18] Sparrow E., Friede M., Sheikh M., Torvaldsen S. (2017). Therapeutic antibodies for infectious diseases. *Bulletin of the World Health Organization*.

[19] Newsome B. W., Ernstoff M. S. (2008). The clinical pharmacology of therapeutic monoclonal antibodies in the treatment of malignancy; have the magic bullets arrived?. *British Journal of Clinical Pharmacology*.

[20] Kung P. C., Goldstein G., Reinherz E. L., Schlossman S. F. (1979). Monoclonal antibodies defining distinctive human T cell surface antigens. *Science*.

[21] Alberts B., Johnson A., Lewis J., Raff M., Roberts K., Walter P. (2002). *Molecular Biology of the Cell*.

[22] Tjandra J. J., Ramadi L., McKenzie I. F. C. (1990). Development of human anti‐murine antibody (HAMA) response in patients. *Immunology & Cell Biology*.

[23] Moi M. K., DeNardo S. J., Meares C. F. (1990). Stable bifunctional chelates of metals used in radiotherapy. *Cancer Research*.

[24] Pendley C., Schantz A., Wagner C. (2003). Immunogenicity of therapeutic monoclonal antibodies. *Current Opinion in Molecular Therapeutics*.

[25] Stern M., Herrmann R. (2005). Overview of monoclonal antibodies in cancer therapy: present and promise. *Critical Review in Oncology/Hematology*.

[26] Morrison S. L., Johnson M. J., Herzenberg L. A., Oi V. T. (1984). Chimeric human antibody molecules: mouse antigen-binding domains with human constant region domains. *Proceedings of the National Acadamy of Sciences of the United States of America*.

[27] LoBuglio A. F., Wheeler R. H., Trang J. (1989). Mouse/human chimeric monoclonal antibody in man: kinetics and immune response. *Proceedings of the National Acadamy of Sciences of the United States of America*.

[28] Brüggemann M., Winter G., Waldmann H., Neuberger M. S. (1989). The immunogenicity of chimeric antibodies. *The Journal of Experimental Medicine*.

[29] Scott S. D. (1998). Rituximab: a new therapeutic monoclonal antibody for non-Hodgkin's lymphoma. *Cancer Practice*.

[30] Osborn M. J., Ma B., Avis S. (2013). High-affinity IgG antibodies develop naturally in Ig-knockout rats carrying germline human IgH/Ig*κ*/Ig*λ* loci bearing the rat CH region. *The Journal of Immunology*.

[31] Jones P. T., Dear P. H., Foote J., Neuberger M. S., Winter G. (1986). Replacing the complementarity-determining regions in a human antibody with those from a mouse. *Nature*.

[32] Riechmann L., Clark M., Waldmann H., Winter G. (1988). Reshaping human antibodies for therapy. *Nature*.

[33] Queen C., Schneider W. P., Selick H. E. (1989). A humanized antibody that binds to the interleukin 2 receptor. *Proceedings of the National Acadamy of Sciences of the United States of America*.

[34] Maneewatch S., Sakolvaree Y., Tapchaisri P. (2009). Humanized-monoclonal antibody against heterologous *Leptospira* infection. *Protein Engineering, Design and Selection*.

[35] Roguska M. A., Pedersen J. T., Keddy C. A. (1994). Humanization of murine monoclonal antibodies through variable domain resurfacing. *Proceedings of the National Acadamy of Sciences of the United States of America*.

[36] Reichert J., Pavlou A. (2004). Monoclonal antibodies market. Market analysis. *Nature Reviews Drug Discovery*.

[37] Buss N. A. P. S., Henderson S. J., McFarlane M., Shenton J. M., De Haan L. (2012). Monoclonal antibody therapeutics: history and future. *Current Opinion in Pharmacology*.

[38] Borrebaeck C. A. K., Danielsson L., Moller S. A. (1988). Human monoclonal antibodies produced by primary *in vitro* immunization of peripheral blood lymphocytes. *Proceedings of the National Acadamy of Sciences of the United States of America*.

[39] Simmons C. P., Bernasconi N. L., Suguitan A. L. (2007). Prophylactic and therapeutic efficacy of human monoclonal antibodies against H5N1 influenza. *PLoS Medicine*.

[40] Chiorazzi N., Wasserman R. L., Kunkel H. G. (1982). Use of Epstein-Barr virus-transformed B cell lines for the generation of immunoglobulin-producing human B cell hybridomas. *The Journal of Experimental Medicine*.

[41] Foung S. K. H., Perkins S., Raubitschek A. (1984). Rescue of human monoclonal antibody production from an EBV-transformed B cell line by fusion to a human-mouse hybridoma. *Journal of Immunological Methods*.

[42] Garzelli C., Puglisi C., Falcone G. (1986). Human monoclonal antibody to purified protein derivative of tuberculin produced by hybrids constructed with Epstein‐Barr virus‐transformed B lymphocytes and mouse myeloma cells. *European Journal of Immunology*.

[43] Kwakkenbos M. J., van Helden P. M., Beaumont T., Spits H. (2016). Stable long-term cultures of self-renewing B cells and their applications. *Immunological Reviews*.

[44] Bruggemann M., Caskey H. M., Teale C. (1989). A repertoire of monoclonal antibodies with human heavy chains from transgenic mice. *Proceedings of the National Acadamy of Sciences of the United States of America*.

[45] Lonberg N., Huszar D. (1995). Human antibodies from transgenic mice. *International Reviews of Immunology*.

[46] Fishwild D. M., O'Donnell S. L., Bengoechea T. (1996). High-avidity human IgG*κ* monoclonal antibodies from a novel strain of minilocus transgenic mice. *Nature Biotechnology*.

[47] Green L. L. (1999). Antibody engineering via genetic engineering of the mouse: XenoMouse strains are a vehicle for the facile generation of therapeutic human monoclonal antibodies. *Journal of Immunological Methods*.

[48] Kuroiwa Y., Kasinathan P., Choi Y. J. (2002). Cloned transchromosomic calves producing human immunoglobulin. *Nature Biotechnology*.

[49] Lonberg N. (2005). Human antibodies from transgenic animals. *Nature Biotechnology*.

[50] Ouisse L. H., Gautreau-Rolland L., Devilder M. C. (2017). Antigen-specific single B cell sorting and expression-cloning from immunoglobulin humanized rats: a rapid and versatile method for the generation of high affinity and discriminative human monoclonal antibodies. *BMC Biotechnology*.

[51] Brüggemann M., Osborn M. J., Ma B. (2015). Human antibody production in transgenic animals. *Archivum Immunologiae et Therapia Experimentalis*.

[52] Nelson A., Dhimolea E., Reichert J. M. (2010). Development trends for human monoclonal antibody therapeutics. *Nature Reviews Drug Discovery*.

[53] Smith G. P. (1985). Filamentous fusion phage: novel expression vectors that display cloned antigens on the virion surface. *Science*.

[54] Hoogenboom H. R. (2005). Selecting and screening recombinant antibody libraries. *Nature Biotechnology*.

[55] Sidhu S. S., Fellouse F. A. (2006). Synthetic therapeutic antibodies. *Nature Chemical Biology*.

[56] Kulkeaw K., Sakolvaree Y., Srimanote P. (2009). Human monoclonal ScFv neutralize lethal Thai cobra, *Naja kaouthia*, neurotoxin. *Journal of Proteomics*.

[57] Boder E. T., Wittrup K. D. (1997). Yeast surface display for screening combinatorial polypeptide libraries. *Nature Biotechnology*.

[58] Daugherty P. S., Olsen M. J., Iverson B. L., Georgiou G. (1999). Development of an optimized expression system for the screening of antibody libraries displayed on the *Escherichia coli* surface. *Protein Engineering, Design and Selection*.

[59] Beerli R. R., Bauer M., Buser R. B. (2008). Isolation of human monoclonal antibodies by mammalian cell display. *Proceedings of the National Acadamy of Sciences of the United States of America*.

[60] Hanes J., Plückthun A. (1997). *In vitro* selection and evolution of functional proteins by using ribosome display. *Proceedings of the National Acadamy of Sciences of the United States of America*.

[61] Hoogenboom H. R., O’Brien P. M., Aitken R. R. (2002). Overview of antibody phage-display technology and its application. *Antibody Phage Display Methods and Protocol*.

[62] Winter G., Griffiths A. D., Hawkins R. E., Hoogenboom H. R. (1994). Making antibodies by phage display technology. *Annual Review of Immunology*.

[63] V BASE: the database of human antibody genes, MRC Center for Protein Engineering. http://vbase.mrc-cpe.cam.ac.uk.

[64] Hamers-Casterman C., Atarhouch T., Muyldermans S. (1993). Naturally occurring antibodies devoid of light chains. *Nature*.

[65] Joosten V., Lokman C., van den Hondel C. A. M. J. J., Punt P. J. (2003). The production of antibody fragments and antibody fusion proteins by yeasts and filamentous fungi. *Microbial Cell Factories*.

[66] Muyldermans S., Atarhouch T., Saldanha J., Barbosa J. A. R. G., Hamers R. (1994). Sequence and structure of V(H) domain from naturally occurring camel heavy chain immunoglobulins lacking light chains. *Protein Engineering, Design and Selection*.

[67] Wu T. T., Johnson G., Kabat E. A. (1993). Length distribution of CDRH3 in antibodies. *Proteins: Structure, Function, and Bioinformatics*.

[68] Harmsen M. M., De Haard H. J. (2007). Properties, production, and applications of camelid single-domain antibody fragments. *Applied Microbiology and Biotechnology*.

[69] Arbabi Ghahroudi M., Desmyter A., Wyns L., Hamers R., Muyldermans S. (1997). Selection and identification of single domain antibody fragments from camel heavy-chain antibodies. *FEBS Letters*.

[70] Muyldermans S. (2001). Single domain camel antibodies: current status. *Journal of Biotechnology*.

[71] Lauwereys M., Ghahroudi M. A., Desmyter A. (1998). Potent enzyme inhibitors derived from dromedary heavy-chain antibodies. *EMBO Journal*.

[72] Jobling S. A., Jarman C., Teh M. M., Holmberg N., Blake C., Verhoeyen M. E. (2003). Immunomodulation of enzyme function in plants by single-domain antibody fragments. *Nature Biotechnology*.

[73] Conrath K. E., Lauwereys M., Galleni M. (2001). *β*-Lactamase inhibitors derived from single-domain antibody fragments elicited in the Camelidae. *Antimicrobial Agents and Chemotherapy*.

[74] Thanongsaksrikul J., Srimanote P., Maneewatch S. (2010). A VHH that neutralizes the zinc metalloproteinase activity of botulinum neurotoxin type A. *The Journal of Biological Chemistry*.

[75] Desmyter A., Spinelli S., Payan F. (2002). Three camelid VHH domains in complex with porcine pancreatic *α*-amylase: inhibition and versatility of binding topology. *The Journal of Biological Chemistry*.

[76] De Genst E., Saerens D., Muyldermans S., Conrath K. (2006). Antibody repertoire development in camelids. *Developmental & Comparative Immunology*.

[77] Revets H., De Baetselier P., Muyldermans S. (2005). Nanobodies as novel agents for cancer therapy. *Expert Opinion on Biological Therapy*.

[78] Cortez-Retamozo V., Backmann N., Senter P. D. (2004). Efficient cancer therapy with a nanobody-based conjugate. *Cancer Research*.

[79] Wesolowski J., Alzogaray V., Reyelt J. (2009). Single domain antibodies: promising experimental and therapeutic tools in infection and immunity. *Medical Microbiology and Immunology*.

[80] Baral T. N., Magez S., Stijlemans B. (2006). Experimental therapy of African trypanosomiasis with a nanobody-conjugated human trypanolytic factor. *Nature Medicine*.

[81] Koch-Nolte F., Reyelt J., Schößow B. (2007). Single domain antibodies from llama effectively and specifically block T cell ecto-ADP-ribosyltransferase ART2.2 *in vivo*. *The FASEB Journal*.

[82] Chavanayarn C., Thanongsaksrikul J., Thueng-in K., Bangphoomi K., Sookrung N., Chaicumpa W. (2012). Humanized-single domain antibodies (VH/VHH) that bound specifically to *Naja kaouthia* phospholipase A2 and neutralized the enzymatic activity. *Toxins*.

[83] Phalaphol A., Thueng-in K., Thanongsaksrikul J. (2013). Humanized-VH/VHH that inhibit HCV replication by interfering with the virus helicase activity. *Journal of Virological Methods*.

[84] Thueng-in K., Thanongsaksrikul J., Srimanote P. (2012). Cell penetrable humanized-VH/VHH that inhibit RNA dependent RNA polymerase (NS5B) of HCV. *PLoS ONE*.

[85] Jittavisutthikul S., Thanongsaksrikul J., Thueng-in K. (2015). Humanized-V_H_H transbodies that inhibit HCV protease and replication. *Viruses*.

[86] Wang L., Jia J., Wang C. (2013). Inhibition of synovitis and joint destruction by a new single domain antibody specific for cyclophilin A in two different mouse models of rheumatoid arthritis. *Arthritis Research Therapy*.

[87] Nelson A. L., Reichert J. M. (2009). Development trends for therapeutic antibody fragments. *Nature Biotechnology*.

[88] Holz J. B. (2012). The TITAN trial - Assessing the efficacy and safety of an anti-von Willebrand factor nanobody in patients with acquired thrombotic thrombocytopenic purpura. *Transfusion and Apheresis Science*.

[89] De Groeve K., Deschacht N., De Koninck C. (2010). Nanobodies as tools for *in vivo* imaging of specific immune cell types. *Journal of Nuclear Medicine: official publication, Society of Nuclear Medicine*.

[90] Vaneycken I., D'huyvetter M., Hernot S. (2011). Immuno-imaging using nanobodies. *Current Opinion in Biotechnology*.

[91] Chakravarty R., Goel S., Cai W. (2014). Nanobody: the ‘magic bullet’ for molecular imaging?. *Theranostics*.

[92] De Meyer T., Muyldermans S., Depicker A. (2014). Nanobody-based products as research and diagnostic tools. *Trends in Biotechnology*.

[93] Cooper G. M. (2000). The Cell: A Molecular Approach. *Transport of Small Molecules*.

[94] Zelphati O., Szoka F. C. (1996). Intracellular distribution and mechanism of delivery of oligonucleotides mediated by cationic lipids. *Pharmaceutical Research*.

[95] Kitazoe M., Murata H., Futami J. (2005). Protein transduction assisted by polyethylenimine-cationized carrier proteins. *The Journal of Biochemistry*.

[96] Derossi D., Joliot A. H., Chassaing G., Prochiantz A. (1994). The third helix of the Antennapedia homeodomain translocates through biological membranes. *The Journal of Biological Chemistry*.

[97] Pooga M., Kut C., Kihlmark M. (2001). Cellular translocation of proteins by transportan. *The FASEB Journal*.

[98] Deshayes S., Morris M. C., Divita G., Heitz F. (2005). Cell-penetrating peptides: tools for intracellular delivery of therapeutics. *Cellular and Molecular Life Sciences*.

[99] Morris M. C., Deshayes S., Simeoni F., Aldrien-Herrada G., Heitz F., Divita G., Langel Ü. (2008). A non-covalent peptide-based carrier for peptide and short interfering RNA delivery. *Cell Penetrating Peptides*.

[100] Heitz F., Morris M. C., Divita G. (2009). Twenty years of cell-penetrating peptides: from molecular mechanisms to therapeutics. *British Journal of Pharmacology*.

[101] Morris M. C., Vidal P., Chaloin L., Heitz F., Divita G. (1997). A new peptide vector for efficient delivery of oligonucleotides into mammalian cells. *Nucleic Acids Research*.

[102] Noguchi H., Matsumoto S. (2006). Protein transduction technology: a novel therapeutic perspective. *Acta Medica Okayama*.

[103] Poungpair O., Pootong A., Maneewatch S. (2010). A human single chain transbody specific to matrix protein (M1) interferes with the replication of influenza a virus. *Bioconjugate Chemistry*.

[104] Dong-Din-On F., Songserm T., Pissawong T. (2015). Cell penetrable human scFv specific to middle domain of matrix protein-1 protects mice from lethal influenza. *Viruses*.

[105] Pujals S., Fernández-Carneado J., López-Iglesias C., Kogan M. J., Giralt E. (2006). Mechanistic aspects of CPP-mediated intracellular drug delivery: relevance of CPP self-assembly. *Biochimica et Biophysica Acta*.

[106] Snyder E. L., Dowdy S. F. (2005). Recent advances in the use of protein transduction domains for the delivery of peptides, proteins and nucleic acids *in vivo*. *Expert Opinion on Drug Delivery*.

[107] Schwarze S. R., Ho A., Vocero-Akbani A., Dowdy S. F. (1999). *In vivo* protein transduction: delivery of a biologically active protein into the mouse. *Science*.

[108] Elliott G., O'Hare P. (1997). Intercellular trafficking and protein delivery by a herpesvirus structural protein. *Cell*.

[109] Noguchi H., Bonner-Weir S., Wei F. Y., Matsushita M., Matsumoto S. (2005). BETA2/NeuroD protein can be transduced into cells due to an arginine- and lysine-rich sequence. *Diabetes*.

[110] Teimoori S., Seesuay W., Jittavisutthikul S. (2016). Human transbodies to VP40 inhibit cellular egress of Ebola virus-like particles. *Biochemical and Biophysical Research Communications*.

[111] Jittavisutthikul S., Seesuay W., Thanongsaksrikul J. (2016). Human transbodies to HCV NS3/4A protease inhibit viral replication and restore host innate immunity. *Frontiers in Immunology*.

[112] Glab-ampai K., Malik A. A., Chulanetra M. (2016). Inhibition of HCV replication by humanized-single domain transbodies to NS4B. *Biochemical and Biophysical Research Communications*.

[113] Webster R. G., Wright S. M., Castrucci M. R., Bean W. J., Kawaoka Y. (1993). Influenza - a model of an emerging virus disease. *Intervirology*.

[114] Lamp R. A., Kung R. M., Knipe D. M., Howley PM P. M., Griffin D. T. (2001). Orthomyxoviridae: the viruses and their replication. *Fields Virology*.

[115] World Health Organization (2005). *H5N1 Avian Influenza: Timeline*.

[116] Suguitan Jr. A. L., Subbarao K., Taboe E. (2006). The Pandemic Threat of Avian Influenza Viruses. *Emerging Viruses in Human Populations*.

[117] Center for Disease Control and Prevention (2009). Update: novel influenza A (H1N1) virus infections worldwide. *Morbidity Mortality Weekly Report*.

[118] Kawano H., Haruyama T., Hayashi Y., Sinoda Y., Sonoda M., Kobayashi N. (2011). Genetic analysis and phylogenetic characterization of pandemic (H1N1) 2009 influenza viruses that found in Nagasaki, Japan. *Japanese Journal of Infectious Diseases*.

[119] Center for Disease Control and Prevention (2010). The 2009 H1N1 pandemic: summary highlights, April 2009–April 2010. http://www.Cdc.gov/h1n1flu/cdcresponse.htm.

[120] Dolin R. (2013). The quadrivalent approach to influenza vaccination. *The Journal of Infectious Diseases*.

[121] Jennings Z., Carter I., McPhie K., Kok J., Dwyer D. E. (2015). Increased prevalence of influenza B/victoria lineage viruses during early stages of the 2015 influenza season in new South Wales, Australia: implications for vaccination and planning. *Eurosurveillance*.

[122] http://www.cdc.gov/flu/avianflu/influenza-a-virus-subtypes.htm

[123] Wu Y., Wu Y., Tefsen B., Shi Y., Gao G. F. (2014). Bat-derived influenza-like viruses H17N10 and H18N11. *Trends in Microbiology*.

[124] http://www.phac-aspc.gc.ca/lab-bio/res/psds-ftss/influenza-grippe-b-c-eng.php

[125] Osterhaus A. D. N. E., Rimmeizwaan G. F., Martina B. E. E., Bestebroer T. M., Fouchier R. A. M. (2000). Influenza B in seals. *Science*.

[126] Ohishi K., Ninomiya A., Kida H. (2002). Serological evidence of transmission of human influenza A and B viruses to Caspian seals (*Phoca caspica*). *Microbiology and Immunology*.

[127] Acha P. N., Szyfres B. (2003). Zoonoses and communicable diseases common to man and animals. *Pan American Health Organization*.

[128] Moa A. M., Muscatello D. J., Turner R. M., MacIntyre C. R. (2017). Epidemiology of influenza B in Australia: 2001-2014 influenza seasons. *Influenza and Other Respiratory Viruses*.

[129] Youzbashi E., Marschall M., Chaloupka I., Meier-Ewert H. (1999). Distribution of influenza C virus infection in dogs and pigs in Bavaria. *Tierarziliche Praxis*.

[130] Ducatez M. F., Pelletier C., Meyer G. (2015). Influenza D virus in cattle, France, 2011–2014. *Emerging Infectious Diseases*.

[131] Zhai S. L., Zhang H., Chen S.-N. (2017). Influenza D virus in animal species in Guangdong Province, Southern China. *Emerging Infectious Diseases*.

[132] Hause B. M., Collin E. A., Liu R. (2014). Characterization of a novel influenza virus in cattle and swine: proposal for a new genus in the Orthomyxoviridae family. *mBio*.

[133] Vasin A., Temkina O., Egorov V., Klotchenko S., Plotnikova M., Kiselev O. (2014). Molecular mechanisms enhancing the proteome of influenza A viruses: an overview of recently discovered proteins. *Virus Research*.

[134] Turrell L., Lyall J. W., Tiley L. S., Fodor E., Vreede F. T. (2013). The role and assembly mechanism of nucleoprotein in influenza A virus ribonucleoprotein complexes. *Nature Communications*.

[135] Li M. L., Rao P., Krug R. M. (2001). The active sites of the influenza cap-dependent endonuclease are on different polymerase subunits. *EMBO Journal*.

[136] Cianci C., Tiley L., Krystal M. (1995). Differential activation of the influenza virus polymerase *via* template RNA binding. *Journal of Virology*.

[137] Li M. L., Ramirez B. C., Krug R. M. (1998). RNA-dependent activation of primer RNA production by influenza virus polymerase: different regions of the same protein subunit constitute the two required RNA-binding sites. *EMBO Journal*.

[138] Gonzalez S., Ortin J. (1999). Distinct regions of influenza virus PB1 polymerase subunit recognize vRNA and cRNA templates. *EMBO Journal*.

[139] Fechter P., Mingay L., Sharps J., Chambers A., Fodor E., Brownlee G. G. (2003). Two aromatic residues in the PB2 subunit of influenza A RNA polymerase are crucial for cap binding. *The Journal of Biological Chemistry*.

[140] Neumann G., Brownlee G. G., Fodor E., Kawaoka Y. (2004). Orthomyxovirus replication, transcription, and polyadenylation. *Current Topics in Microbiology and Immunology*.

[141] Fodor E., Crow M., Mingay L. J. (2002). A single amino acid mutation in the PA subunit of the influenza virus RNA polymerase inhibits endonucleolytic cleavage of capped RNAs. *Journal of Virology*.

[142] Huarte M., Falcón A., Nakaya Y., Ortín J., García-Sastre A., Nietol A. (2003). Threonine 157 of influenza virus PA polymerase subunit modulates RNA replication in infectious viruses. *Journal of Virology*.

[143] Kawaguchi A., Naito T., Nagata K. (2005). Involvement of influenza virus PA subunit in assembly of functional RNA polymerase complexes. *Journal of Virology*.

[144] Chen W., Calvo P. A., Malide D. (2001). A novel influenza A virus mitochondrial protein that induces cell death. *Nature Medicine*.

[145] Wise H. M., Foeglein A., Sun J. (2009). A complicated message: identification of a novel PB1-related protein translated from influenza A virus segment 2 mRNA. *Journal of Virology*.

[146] Jagger B. W., Wise H. M., Kash J. C. (2012). An overlapping protein-coding region in influenza A virus segment 3 modulates the host response. *Science*.

[147] Shi M., Jagger B. W., Wise H. M., Digard P., Holmes E. C., Taubenberger J. K. (2012). Evolutionary conservation of the PA-X open reading frame in segment 3 of influenza a virus. *Journal of Virology*.

[148] Bavagnoli L., Cucuzza S., Campanini G. (2015). The novel influenza A virus protein PA-X and its naturally deleted variant show different enzymatic properties in comparison to the viral endonuclease PA. *Nucleic Acids Research*.

[149] Hayashi T., MacDonald L. A., Takimoto T. (2015). Influenza A virus protein PA-X contributes to viral growth and suppression of the host antiviral and immune responses. *Journal of Virology*.

[150] Muramoto Y., Noda T., Kawakami E., Akkina R., Kawaokaa Y. (2013). Identification of novel influenza A virus proteins translated from PA mRNA. *Journal of Virology*.

[151] Copeland C. S., Doms R. W., Bolzau E. M., Webster R. G., Helenius A. (1986). Assembly of influenza hemagglutinin trimers and its role in intracellular transport. *The Journal of Cell Biology*.

[152] Gething M. J., McCammon K., Sambrook J. (1986). Expression of wild-type and mutant forms of influenza hemagglutinin: The role of folding in intracellular transport. *Cell*.

[153] Klenk H. D., Rott R., Orlich M., Bloedorn J. (1975). Activation of influenza A viruses by trypsin treatment. *Virology*.

[154] Lazarowitz S. G., Choppin P. W. (1975). Enhancement of the infectivity of influenca A and B viruses by proteolytic cleavage of the hemagglutinin polypeptide. *Virology*.

[155] Skehel J. J., Wiley D. C. (2000). Receptor binding and membrane fusion in virus entry: the influenza hemagglutinin. *Annual Review of Biochemistry*.

[156] Li S., Sieben C., Ludwig K. (2014). pH-controlled two-step uncoating of influenza virus. *Biophysical Journal*.

[157] Dowdle W. R., Downie J. C., Laver W. G. (1974). Inhibition of virus release by antibodies to surface antigens of influenza viruses. *Journal of Virology*.

[158] Matrosovich M. N., Matrosovich T. Y., Gray T., Roberts N. A., Klenk H. D. (2004). Neuraminidase is important for the initiation of influenza virus infection in human airway epithelium. *Journal of Virology*.

[159] Huang I. C., Li W., Sui J., Marasco W., Choe H., Farzan M. (2008). Influenza A virus neuraminidase limits viral superinfection. *Journal of Virology*.

[160] Dubois J., Terrier O., Rosa-Calatrava M. (2014). Influenza viruses and mRNA splicing: doing more with less. *mBio*.

[161] Zhao H., Ekström M., Garoff H. (1998). The M1 and NP proteins of influenza A virus form homo- but not heterooligomeric complexes when coexpressed in BHK-21 cells. *Journal of General Virology*.

[162] Schulze I. T. (1972). The structure of influenza virus. II. A model based on the morphology and composition of subviral particles. *Virology*.

[163] Noton S. L., Medcalf E., Fisher D., Mullin A. E., Elton D., Digard P. (2007). Identification of the domains of the influenza A virus M1 matrix protein required for NP binding, oligomerization and incorporation into virions. *Journal of General Virology*.

[164] Ali A., Avalos R. T., Ponimaskin E., Nayak D. P. (2000). Influenza virus assembly: effect of influenza virus glycoproteins on the membrane association of M1 protein. *Journal of Virology*.

[165] Gómez-Puertas P., Albo C., Pérez-Pastrana E., Vivo A., Portela A. (2000). Influenza virus matrix protein is the major driving force in virus budding. *Journal of Virology*.

[166] Zhang K., Wang Z., Liu X. (2012). Dissection of influenza A virus M1 protein: pH-dependent oligomerization of N-terminal domain and dimerization of C-terminal domain. *PLoS ONE*.

[167] Nayak D. P., Hui E. K. W. (2002). Assembly and morphogenesis of influenza viruses. *Recent Researches in Developmental Virology*.

[168] Bui M., Whittaker G., Helenius A. (1996). Effect of M1 protein and low pH on nuclear transport of influenza virus ribonucleoproteins. *Journal of Virology*.

[169] Yasuda J., Nakada S., Kato A., Toyoda T., Ishihama A. (1993). Molecular assembly of influenza virus: association of the NS2 protein with virion matrix. *Virology*.

[170] Akarsu H., Burmeister W. P., Petosa C. (2003). Crystal structure of the M1 protein-binding domain of the influenza A virus nuclear export protein (NEP/NS2). *EMBO Journal*.

[171] Shimizu T., Takizawa N., Watanabe K., Nagata K., Kobayashi N. (2011). Crucial role of the influenza virus NS2 (NEP) C-terminal domain in M1 binding and nuclear export of vRNP. *FEBS Letters*.

[172] Whittaker G., Bui M., Helenius A. (1996). Nuclear trafficking of influenza virus ribonucleoproteins in heterokaryons. *Journal of Virology*.

[173] Whittaker G. (1996). The role of nuclear import and export in influenza virus infection. *Trends in Cell Biology*.

[174] Nieva J. L., Madan V., Carrasco L. (2012). Viroporins: structure and biological functions. *Nature Reviews Microbiology*.

[175] Helenius A. (1992). Unpacking the incoming influenza virus. *Cell*.

[176] Gannagé M., Dormann D., Albrecht R. (2009). Matrix protein 2 of influenza A virus blocks autophagosome fusion with lysosomes. *Cell Host & Microbe*.

[177] Ciampor F., Bayley P. M., Nermut M. V., Hirst E. M. A., Sugrue R. J., Hay A. J. (1992). Evidence that the amantadine-induced, M2-mediated conversion of influenza A virus hemagglutinin to the low pH conformation occurs in an acidic trans golgi compartment. *Virology*.

[178] Rossman J. S., Lamb R. A. (2011). Influenza virus assembly and budding. *Virology*.

[179] Wise H. M., Hutchinson E. C., Jagger B. W. (2012). Identification of a novel splice variant form of the influenza A virus M2 ion channel with an antigenically distinct ectodomain. *PLoS Pathogens*.

[180] Hale B. G., Batty I. H., Downes C. P., Randall R. E. (2008). Binding of influenza A virus NS1 protein to the inter-SH2 domain of p85*β* suggests a novel mechanism for phosphoinositide 3-kinase activation. *The Journal of Biological Chemistry*.

[181] Lamb R. A., Lai C. J. (1980). Sequence of interrupted and uninterrupted mRNAs and cloned DNA coding for the two overlapping nonstructural proteins of influenza virus. *Cell*.

[182] Kim S. H., Samal S. K. (2010). Inhibition of host innate immune responses and pathogenicity of recombinant Newcastle disease viruses expressing NS1 genes of influenza A viruses. *Journal of General Virology*.

[183] Lin D., Lan J., Zhang Z. (2007). Structure and function of the NS1 protein of influenza A virus. *Acta Biochimica et Biophysica Sinica*.

[184] Min J. Y., Krug R. M. (2006). The primary function of RNA binding by the influenza A virus NS1 protein in infected cells: inhibiting the 2′-5′ oligo (A) synthetase/RNase L pathway. *Proceedings of the National Acadamy of Sciences of the United States of America*.

[185] Min J. Y., Li S., Sen G. C., Krug R. M. (2007). A site on the influenza A virus NS1 protein mediates both inhibition of PKR activation and temporal regulation of viral RNA synthesis. *Virology*.

[186] Silverman R. H. (2007). Viral encounters with 2′,5′-oligoadenylate synthetase and RNase L during the interferon antiviral response. *Journal of Virology*.

[187] Hale B. G., Randall R. E., Ortin J., Jackson D. (2008). The multifunctional NS1 protein of influenza A viruses. *Journal of General Virology*.

[188] Nemeroff M. E., Barabino S. M. L., Li Y., Keller W., Krug R. M. (1998). Influenza virus NS1 protein interacts with the cellular 30 kDa subunit of CPSF and inhibits 3′ end formation of cellular pre-mRNAs. *Molecular Cell*.

[189] Chen Z., Li Y., Krug R. M. (1999). Influenza A virus NS1 protein targets poly(A)-binding protein II of the cellular 3′-end processing machinery. *EMBO Journal*.

[190] Aragon T., De La Luna S., Novoa I., Carrasco L., Ortin J., Nieto A. (2000). Eukaryotic translation initiation factor 4GI is a cellular target for NS1 protein, a translational activator of influenza virus. *Molecular and Cellular Biology*.

[191] Burgui I., Aragón T., Ortín J., Nieto A. (2003). PABP1 and eIF4GI associate with influenza virus NS1 protein in viral mRNA translation initiation complexes. *Journal of General Virology*.

[192] Ong L. L., Chan G. F. (2012). Mini review: a glimpse of nonstructural protein 1 of influenza A H1N1. *Insight Pathology*.

[193] Fernandez-Sesma A., Marukian S., Ebersole B. J. (2006). Influenza virus evades innate and adaptive immunity *via* the NS1 protein. *Journal of Virology*.

[194] O'Neill R. E., Talon J., Palese P. (1998). The influenza virus NEP (NS2 protein) mediates the nuclear export of viral ribonucleoproteins. *EMBO Journal*.

[195] Robb N. C., Smith M., Vreede F. T., Fodor E. (2009). NS2/NEP protein regulates transcription and replication of the influenza virus RNA genome. *Journal of General Virology*.

[196] Selman M., Dankar S. K., Forbes N. E., Jia J. J., Brown E. G. (2012). Adaptive mutation in influenza A virus non-structural gene is linked to host switching and induces a novel protein by alternative splicing. *Emerging Microbes and Infections*.

[197] Clifford M., Twigg J., Upton C. (2009). Evidence for a novel gene associated with human influenza A viruses. *Virology Journal*.

[198] Sabath N., Morris J. S., Graur D. (2011). Is there a twelfth protein-coding gene in the genome of influenza A? A selection-based approach to the detection of overlapping genes in closely related sequences. *Journal of Molecular Evolution*.

[199] Zhirnov O. P., Klenk H. D. (2010). Integration of influenza A virus NSP gene into baculaovirus genome and its expression in insect cell. *Voprosy Virusologii*.

[200] Bridges C. B., Kuehnert M. J., Hall C. B. (2003). Transmission of influenza: implications for control in health care settings. *Clinical Infectious Diseases*.

[201] Cohen M., Zhang X. Q., Senaati H. P. (2013). Influenza A penetrates host mucus by cleaving sialic acids with neuraminidase. *Virology Journal*.

[202] Taubenberger J. K., Morens D. M. (2008). The pathology of influenza virus infections. *Annual Review of Pathology: Mechanisms of Disease*.

[203] Van der Sluijs K. F., Van der Poll T., Lutter R., Juffermans N. P., Schultz M. J. (2010). Bench-to-bedside review: bacterial pneumonia with influenza—pathogenesis and clinical implications. *Critical Care*.

[204] Committee on Infectious Disease (2015). Recommendations for prevention and control of influenza in children. *Pediatrics*.

[205] Jefferson T., Jones M. A., Doshi P. (2012). Neuraminidase inhibitors for preventing and treating influenza in healthy adults and children. *Cochrane Database of Systematic Reviews*.

[206] Fiore F. A. D., Gubareva L., Bresee S. L., Uyeki M. T. (2011). Antiviral agents for treatment and chemoprophylaxis of influenza. *Morbidity Mortality Weekly Report*.

[207] Samson M., Pizzorno A., Abed Y., Boivin G. (2013). Influenza virus resistance to neuraminidase inhibitors. *Antiviral Research*.

[208] Dong G., Peng C., Luo J. (2015). Adamantane-resistant influenza A viruses in the world (1902–2013): frequency and distribution of M2 gene mutations. *PLoS ONE*.

[209] Sheu T. G., Fry A. M., Garten R. J. (2011). Dual resistance to adamantanes and oseltamivir among seasonal influenza A(H1N1) viruses: 2008–2010. *The Journal of Infectious Diseases*.

[210] Dolin R., Reichman R. C., Madore H. P., Maynard R., Linton P. N., Webber-Jones J. (1982). A controlled trial of amantadine and rimantadine in the prophylaxis of influenza A infection. *The New England Journal of Medicine*.

[211] Monto A. S., McKimm-Breschkin J. L., Macken C. (2006). Detection of influenza viruses resistant to neuraminidase inhibitors in global surveillance during the first 3 years of their use. *Antimicrobial Agents and Chemotherapy*.

[212] Spanakis N., Pitiriga V., Gennimata V., Tsakris A. (2014). A review of neuraminidase inhibitor susceptibility in influenza strains. *Expert Review of Anti-infective Therapy*.

[213] WHO Weekly epidemiological record relevé épidémiologique hebdomadaire.

[214] Gasparini R., Amicizia D., Lai P. L., Bragazzi N. L., Panatto D. (2014). Compounds with anti-influenza activity: present and future of strategies for the optimal treatment and management of influenza Part II: Future compounds against influenza virus. *Journal of Preventive Medicine and Hygiene*.

[215] Król E., Rychłowska M., Szewczyk B. (2014). Antivirals - current trends in fighting influenza. *Acta Biochimica Polonica*.

[216] Baras B., Bouveret N., Devaster J.-M. (2008). A vaccine manufacturer's approach to address medical needs related to seasonal and pandemic influenza viruses.. *Influenza and Other Respiratory Viruses*.

[217] Chiu C. (2016). Seasonal influenza vaccines and hurdles to mutual protection. *Clinical Microbiology and Infection*.

[218] Franceschini F., Bottau P., Caimmi S. (2015). Vaccination in children with allergy to nonactive vaccine components. *Clinical and Translational Medicine*.

[219] https://www.healthline.com/health/cold-flu/flu-shot-ingredients

[220] Centers for Disease Control and Prevention (2017). *National Center for Immunization and Respiratory Diseases (NCIRD)*.

[221] Perdue M. L., Arnold F., Li S. (2011). The future of cell culture-based influenza vaccine production. *Expert Review of Vaccines*.

[222] Wong S. S., Webby R. J. (2013). Traditional and new influenza vaccines. *Clinical Microbiology Reviews*.

[223] Treanor J. J., Tierney E. L., Zebedee S. L., Lamb R. A., Murphy B. R. (1990). Passively transferred monoclonal antibody to the M2 protein inhibits influenza A virus replication in mice. *Journal of Virology*.

[224] Shriver Z., Trevejo J. M., Sasisekharan R. (2015). Antibody-based strategies to prevent and treat influenza. *Frontiers in Immunology*.

[225] Smirnov Y. A., Lipatov A. S., Gitelman A. K., Claas E. C. J., Osterhaus A. D. M. E. (2000). Prevention and treatment of bronchopneumonia in mice caused by mouse-adapted variant of avian H5N2 influenza A virus using monoclonal antibody against conserved epitope in the HA stem region. *Archives of Virology*.

[226] Renegar K. B., Small P. A., Boykins L. G., Wright P. F. (2004). Role of IgA *versus* IgG in the control of influenza viral infection in the murine respiratory tract. *The Journal of Immunology*.

[227] Luke T. C., Kilbane E. M., Jackson J. L., Hoffman S. L. (2006). Meta-analysis: convalescent blood products for Spanish influenza pneumonia: a future H5N1 treatment?. *Annals of Internal Medicine*.

[228] Zhou B., Zhong N., Guan Y. (2007). Treatment with convalescent plasma for influenza A (H5N1) infection. *The New England Journal of Medicine*.

[229] Kalenik B., Sawicka R., Góra-Sochacka A., Sirko A. (2014). Influenza prevention and treatment by passive immunization. *Acta Biochimica Polonica*.

[230] Sparrow E., Friede M., Sheikh M., Torvaldsen S., Newall A. T. (2016). Passive immunization for influenza through antibody therapies, a review of the pipeline, challenges and potential applications. *Vaccine*.

[231] Chan-Hui P. Y., Swiderek K. M. (2016). Immunological considerations for developing antibody therapeutics for influenza A. *Human Vaccines & Immunotherapeutics*.

[232] Grandea A. G., Olsen O. A., Cox T. C. (2010). Human antibodies reveal a protective epitope that is highly conserved among human and nonhuman influenza A viruses. *Proceedings of the National Acadamy of Sciences of the United States of America*.

[233] Ekiert D. C., Friesen R. H. E., Bhabha G. (2011). A highly conserved neutralizing epitope on group 2 influenza A viruses. *Science*.

[234] Baranovich T., Jones J. C., Russier M. (2016). The hemagglutinin stem-binding monoclonal antibody VIS410 controls influenza virus-induced acute respiratory distress syndrome. *Antimicrobial Agents and Chemotherapy*.

[235] Marjuki H., Mishin V. P., Chai N. (2016). Human monoclonal antibody 81.39a effectively neutralizes emerging influenza A viruses of group 1 and 2 hemagglutinins. *Journal of Virology*.

[236] Ramos E. L., Mitcham J. L., Koller T. D. (2015). Efficacy and safety of treatment with an anti-M2e monoclonal antibody in experimental human influenza. *The Journal of Infectious Diseases*.

[237] Ochiai H., Kurokawa M., Matsui S. (1992). Infection enhancement of influenza A NWS virus in primary murine macrophages by anti-hemagglutinin monoclonal antibody. *Journal of Medical Virology*.

[238] Dutry I., Yen H., Lee H., Peiris M., Jaume M. (2011). Antibody-dependent enhancement (ADE) of infection and its possible role in the pathogenesis of influenza. *BMC Proceedings*.

[239] Khurana S., Loving C. L., Manischewitz J. (2013). Vaccine-induced anti-HA2 antibodies promote virus fusion and enhance influenza virus respiratory disease. *Science Translational Medicine*.

[240] Skowronski D. M., de Serres G., Crowcroft N. S. (2010). Association between the 2008-09 seasonal influenza vaccine and pandemic H1N1 illness during spring-summer 2009: four observational studies from Canada. *PLoS Medicine*.

[241] Kobinger G. P., Meunier I., Patel A. (2010). Assessment of the efficacy of commercially available and candidate vaccines against a pandemic H1N1 2009 virus. *The Journal of Infectious Diseases*.

[242] Rodríguez-Paredes M., Esteller M. (2011). Cancer epigenetics reaches mainstream oncology. *Nature Medicine*.

[243] Maneewatch S., Thanongsaksrikul J., Songserm T. (2009). Human single-chain antibodies that neutralize homologous and heterologous strains and clades of influenza A virus subtype H5N1. *Antiviral Therapy*.

[244] Pissawong T., Maneewatch S., Thueng-in K. (2013). Human monoclonal ScFv that bind to different functional domains of M2 and inhibit H5N1 influenza virus replication. *Virology Journal*.

[245] Yodsheewan R., Maneewatch S., Srimanote P. (2013). Human monoclonal ScFv specific to NS1 protein inhibits replication of influenza viruses across types and subtypes. *Antiviral Research*.

[246] Sha B., Luo M. (1997). Structure of a bifunctional membrane-RNA binding protein, influenza virus matrix protein M1. *Nature Structural & Molecular Biology*.

[247] Harris A., Forouhar F., Qiu S., Sha B., Luo M. (2001). The crystal structure of the influenza matrix protein M1 at neutral pH: M1–M1 protein interfaces can rotate in the oligomeric structures of M1. *Virology*.

[248] Zvonarjev A. Y., Ghendon Y. Z. (1980). Influence of membrane (M) protein on influenza A virus virion transcriptase activity *in vitro* and its susceptibility to rimantadine. *Journal of Virology*.

[249] Ye Z., Baylor N. W., Wagner R. R. (1989). Transcription-inhibition and RNA-binding domains of influenza A virus matrix protein mapped with anti-idiotypic antibodies and synthetic peptides. *Journal of Virology*.

[250] Ye Z., Robinson D., Wagner R. R. (1995). Nucleus-targeting domain of the matrix protein (M1) of influenza virus. *Journal of Virology*.

[251] Perez D. R., Donis R. O. (1998). The matrix 1 protein of influenza A virus inhibits the transcriptase activity of a model influenza reporter genome *in vivo*. *Virology*.

[252] Glab-ampai K., Chulanetra M., Malik A. A. (2017). Human single chain-transbodies that bound to domain-I of non-structural protein 5A (NS5A) of hepatitis C virus. *Scientific Reports*.

[253] Kohler H., Paul S. (1998). Superantibody activities: new players in innate and adaptive immune responses. *Trends in Immunology*.

[254] Kim J. Y., Kim Y. G., Lee G. M. (2012). CHO cells in biotechnology for production of recombinant proteins: Current state and further potential. *Applied Microbiology and Biotechnology*.

